# New radiometric ^40^Ar–^39^Ar dates and faunistic analyses refine evolutionary dynamics of Neogene vertebrate assemblages in southern South America

**DOI:** 10.1038/s41598-021-89135-1

**Published:** 2021-05-10

**Authors:** Francisco J. Prevosti, Cristo O. Romano, Analía M. Forasiepi, Sidney Hemming, Ricardo Bonini, Adriana M. Candela, Esperanza Cerdeño, M. Carolina Madozzo Jaén, Pablo E. Ortiz, François Pujos, Luciano Rasia, Gabriela I. Schmidt, Matias Taglioretti, Ross D. E. MacPhee, Ulyses F. J. Pardiñas

**Affiliations:** 1 Museo de Ciencias Antropológicas y Naturales, Universidad Nacional de La Rioja (UNLaR), Av. Luis M. de La Fuente S/N, 5300 La Rioja, Argentina; 2 Consejo Nacional de Investigaciones Científicas y Técnicas (CONICET), Buenos Aires, Argentina; 3 Grupo Paleobiología y Paleoecología, IANIGLA, CCT-CONICET-Mendoza, 5500 Mendoza, Argentina; 4 Lamont-Doherty Earth Observatory, University of Columbia—Earth Institute, Palisades, NY 10964-8000 USA; 5 Instituto de Investigaciones Arqueológicas y Paleontológicas del Cuaternario Pampeano (INCUAPA), CONICET, 7400 Olavarría, Buenos Aires Argentina; 6 División Paleontología Vertebrados, Museo de La Plata, Facultad de Ciencias Naturales y Museo, Universidad Nacional de La Plata, 1900 La Plata, Buenos Aires Argentina; 7 INSUGEO-Facultad de Ciencias Naturales E IML, Universidad Nacional de Tucumán, 4000 San Miguel de Tucumán, Tucumán Argentina; 8 Museo Paleontológico Egidio Feruglio, 9100 Trelew, Chubut Argentina; 9 Laboratorio de Paleontología de Vertebrados, Centro de Investigación Científica y de Transferencia Tecnológica a La Producción (CONICET-Prov. ER-UADER), 3105 Diamante, Entre Ríos Argentina; 10 Museo Municipal de Ciencias Naturales “Lorenzo Scaglia”, 7600 Mar del Plata, Buenos Aires Argentina; 11 Observatorio Patrimonio Arqueológico y Paleontológico (OPAP), Litoral Atlántico Norte (LAN), 7600 Mar del Plata, Buenos Aires Argentina; 12 Geología Costera y Paleoecología—IGCYC FCEYN/CIC—Universidad Nacional de Mar del Plata, 7600 Mar del Plata, Buenos Aires Argentina; 13 Department of Mammalogy, American Museum of Natural History, New York, NY 10024-5102 USA; 14 Instituto de Diversidad y Evolución Austral (IDEAUS), CENPAT, CONICET, 9120 Puerto Madryn, Chubut Argentina; 15 Associate Researcher of the Instituto Nacional de Biodiversidad (INABIO), Quito, Ecuador

**Keywords:** Palaeontology, Palaeontology

## Abstract

The vertebrate fossil record of the Pampean Region of Argentina occupies an important place in South American vertebrate paleontology. An abundance of localities has long been the main basis for constructing the chronostratigraphical/geochronological scale for the late Neogene–Quaternary of South America, as well as for understanding major patterns of vertebrate evolution, including the Great American Biotic Interchange. However, few independently-derived dates are available for constraining this record. In this contribution, we present new ^40^Ar/^39^Ar dates on escorias (likely the product of meteoric impacts) from the Argentinean Atlantic coast and statistically-based biochronological analyses that help to calibrate Late Miocene–Pliocene Pampean faunal successions. For the type areas of the Montehermosan and Chapadmalalan Ages/Stages, our results delimit their age ranges to 4.7–3.7 Ma and ca. 3.74–3.04 Ma, respectively. Additionally, from Buenos Aires Province, dates of 5.17 Ma and 4.33 Ma were recovered for “Huayquerian” and Montehermosan faunas. This information helps to better calibrate important first appearances of allochthonous taxa in South America, including one of the oldest records for procyonids (7.24–5.95 Ma), cricetids (6.95–5.46 Ma), and tayassuids (> 3.74 Ma, oldest high-confidence record). These results also constrain to ca. 3 Ma the last appearances of the autochthonous sparassodonts, as well as terror birds of large/middle body size in South America. South American faunal turnover during the late Neogene, including Late Pliocene extinctions, is interpreted as a consequence of knock-on effects from global climatic changes and initiation of the icehouse climate regime.

## Introduction

**Figure 1:**
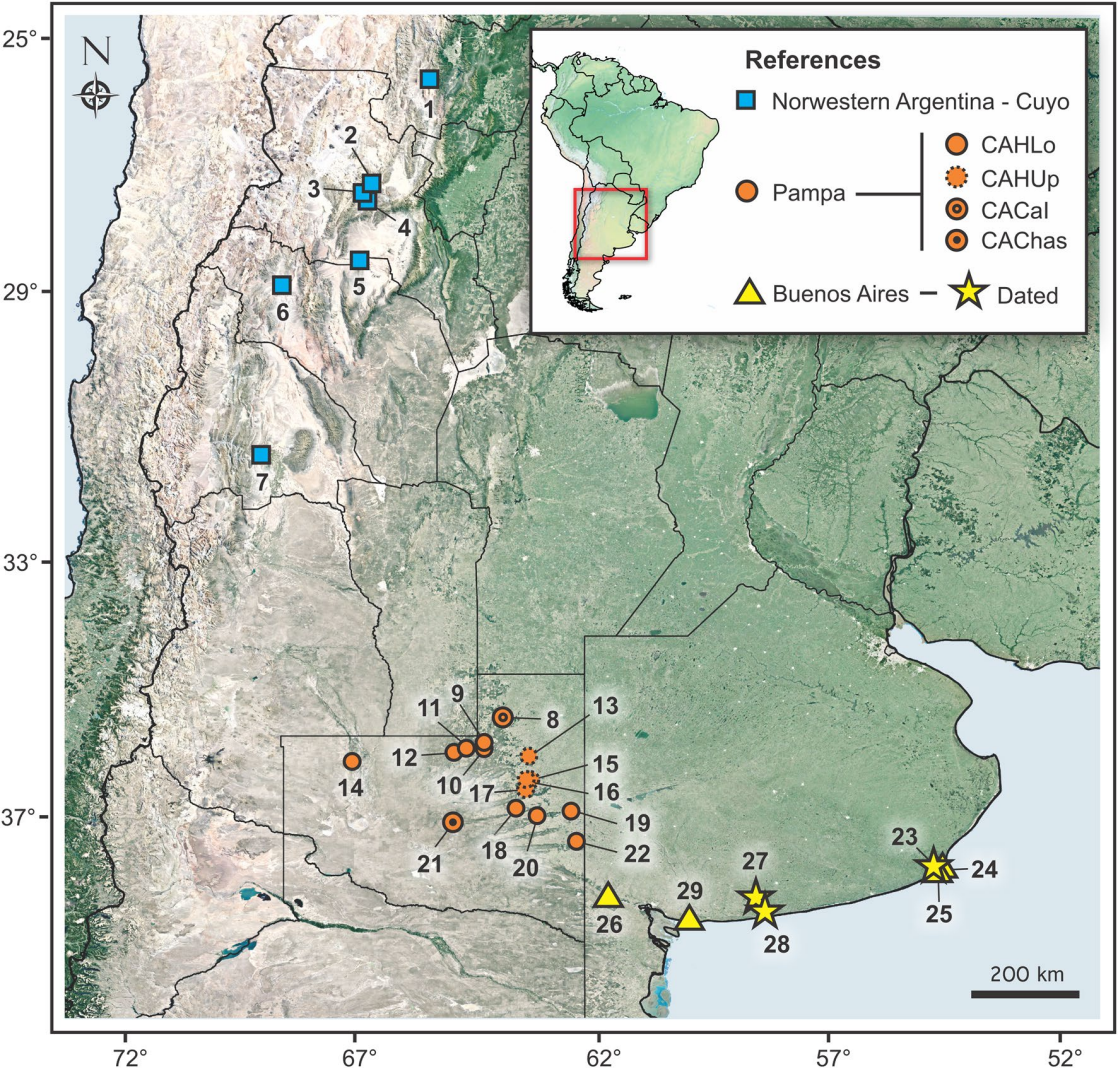
Map showing referenced localities and faunas. 1: Valle del Tonco (PPint); 2: Villavil (AndUp, AndLo, Jar); 3: Puerta de Corral Quemado (AndUp, AndLo, Jar); 4: San Fernando (AndUp, AndLo); 5: El Degolladito (Sal); 6: Quebrada de La Troya (TN); 7: Loma de las Tapias (LTA); 8: Caleufú (CACal); 9: Estancia Ré (CAHLo); 10: Puesto Colorado (CAHLo); 11: Loventué (CAHLo); 12: Telén (CAHLo); 13: El Guanaco (CAHUp); 14: Algarrobo del Águila (CAHLo); 15: Barrancas Coloradas (CAHUp); 16: Bajo Giuliani (CAHUp); 17: Don Mariano (CAHUp); 18: Quehué (CAHLo); 19: Salinas Grandes de Hidalgo (CAHLo); 20: Laguna Chillhué (CAHLo); 21: Cerro La Bota (CAChas); 22: Guatraché (CAHLo); 23: Chapadmalal (Chap); 24: Punta San Andrés (SAnd); 25: Punta Vorohué (Voro); 26: Arroyo Chasicó (Chas); 27: Cascada Grande (QSUp); 28: Paso del Halcón (QSLo); 29: Farola de Monte Hermoso (MH). Localities at La Pampa and Buenos Aires comprise the Pampean Region. The map was generated with QGIS 3.18 (freely available online: https://www.qgis.org/en/site/).

The paleontological significance of the Pampean Region of central Argentina, southern South America (Fig. 1) first attracted attention in the late eighteenth century, when the remains of the giant ground sloth *Megatherium americanum* were discovered. Later, the fossil mammal collections made by Alcide d’Orbigny and Charles Darwin in this area were influential in the development of their ideas about evolution. Since then, and especially during recent decades, new discoveries and innovative techniques in data collection and interpretation^1–5^ have expanded our knowledge of the vertebrate fossil records of the Pampean and nearby regions. As valuable as these new studies are, their impact has been dampened by limitations in the chronological framework available for this part of the southern cone.

**Figure 2:**
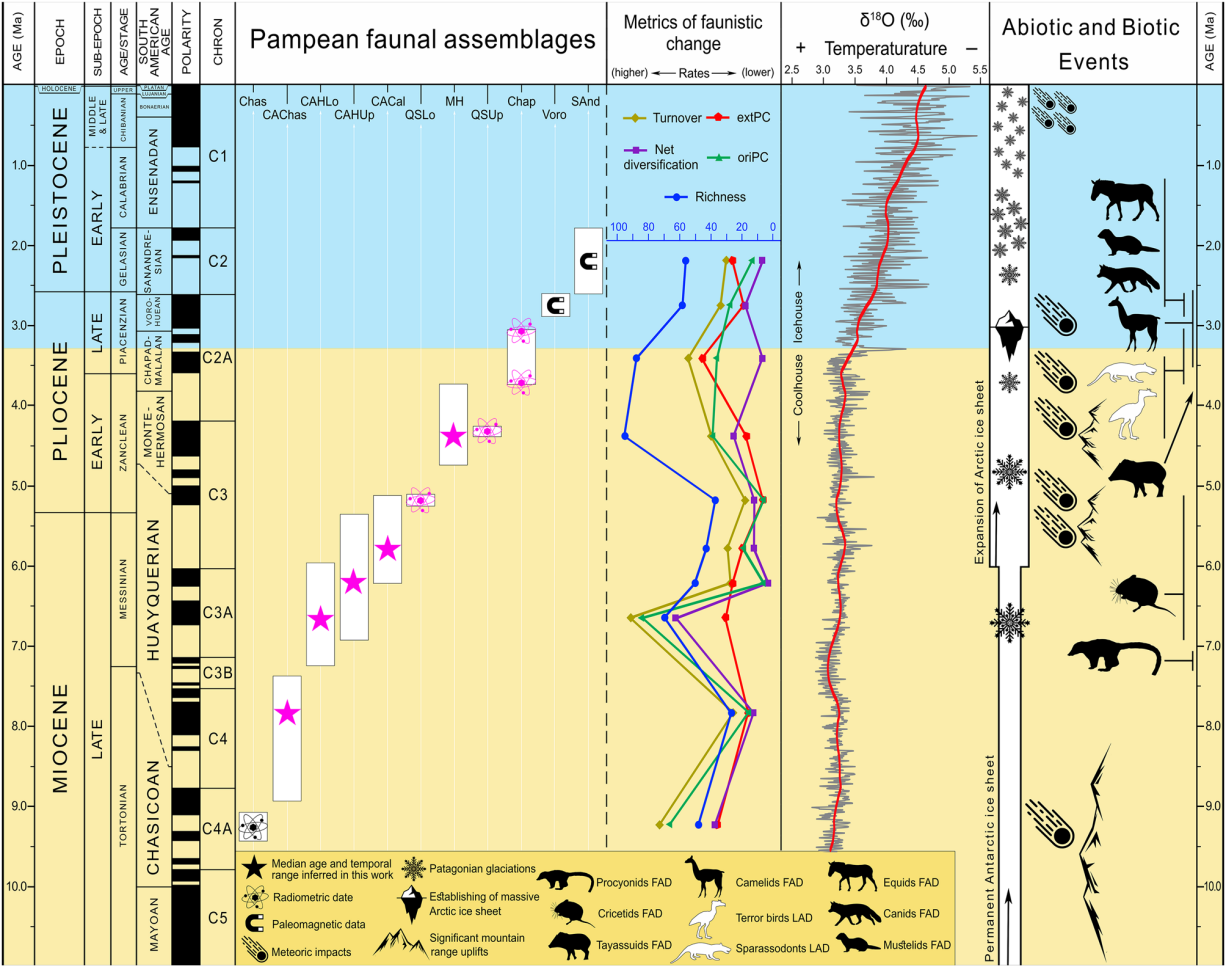
Temporal distribution of investigated faunas and their diversity patterns through time, in correlation with different relevant environmental and faunistic events. Richness, *extPC* per capita extinction, *oriPC* per capita origination. Yellow background: coolhouse climate regime; blue background: icehouse climate regime. Black silhouette: first appearance datum (FAD) of North American immigrants in southern South America; white silhouette: last appearance datum (LAD) of autochthonous lineages in South America. New dates and chronological age inferences are in pink. Temperature curve from ref.^89^. Atom icon was taken and modified from commons.wikimedia.org, designed by Indolences; magnet, snowflake, iceberg and mountains icons were taken and modified from pixabay.com, designed by OpenClipart-Vectors (first two), madartzgraphics, and Radoan_tanvir respectively; meteorite icon was taken and modified from thenounproject.com, designed by icon 54; and representative silhouettes of camelids, equids, procyonids, cricetids, tayassuids, sparassodonts, terror birds, mustelids and canids were taken and modified from philopic.org; and were designed by Steven Traver (first two), RS, uncredited designer, An Ignorant Atheist, Zimices, and Ferran Sayol (last three) respectively. All icons and silhouettes are licensed under Creative Commons Attribution 4.0 Unported license (http://creativecommons.org/licenses/by/4.0/).

Until very recently, age determinations for Late Neogene–Quaternary Pampean fossil vertebrate records, which are overwhelmingly mammalian, were mostly inferred through classical biostratigraphic or biochronological techniques (i.e., seriating fossil assemblages based on specific index taxa, including in a broad sense South American Land Mammal Ages or SALMAs^6^). The well-investigated Pampean fossil associations, originally worked out by Ameghino, Kraglievich, and Pascual and their collaborators, became the essential basis for making continent-wide faunal correlations within the Age/Stage framework^6–8^, nowadays using paleomagnetic and radioisotopic dating when available (Fig. 2). However, in many parts of South America independent geochronological dating is still inadequate for temporally resolving Neogene faunal successions, which hampers a solid understanding of how such turnovers might correlate with global events. This applies to the Pampean Region as well, which is still used for continent-wide faunal correlations^1,6–14^. Although Neogene and Quaternary sediments in this area sometimes have interspersed lenses of Andean tephras, they are otherwise mainly loessoid. “Escorias” may help to change this situation.

Briefly, escorias or pampasites are vesiculated glassy rock fragments, superficially similar to volcanic scoriae (hence the name) but in fact the likely product of extraterrestrial impacts^15–17^; but see ref.^18^. They may be dated isotopically using ^40^Ar/^39^Ar techniques (see Material and Methods^15,16,19^; but see ref.^18^), alone or in combination with paleomagnetic information if available^19,20^. In this contribution we present new ^40^Ar/^39^Ar dates for escorias collected along the Pampean Atlantic coast from the “Irene” and Chapadmalal Formations, neither of which have been adequately dated radioisotopically, see also^15^.

This dataset, combined with other results and statistically-based biochronological analyses, serves as a basis for calibrating Late Miocene–Pliocene faunal succession in the Pampean Region. These results permit both a better determination of first (FAD) and last (LAD) appearance datums for certain taxa involved in early phases of the Great American Biotic Interchange (GABI) and a more quantitative evaluation of mammalian faunal turnover and patterns of evolution in this area than was previously possible.

## Results

### New escoria ^40^Ar/^39^Ar dates and implications for Age/Stage ranges

We dated seven escoria samples from four distinct levels of two Neogene stratigraphic units conforming to the upper and lower levels of the “Irenean” and the Chapadmalal Fm (Fig. 1; for all abbreviations see Table 1). All samples yielded plateaus, defined as > 50% of ^39^Ar released with at least three steps overlapping at two sigmas (Supplementary Table S1, Fig. S1–S3). The “Irenean” fauna is poorly studied in comparison to other Pampean faunas. This unit was initially regarded as a mix of faunas of different ages^21^; its richer late assemblage (QSUp) was interpreted as older than Chap^22^ and more comparable to MH, while the older QSLo was thought to be more similar to the “Huayquerian” assemblage^21,22^.

**Table 1:** Geochronological dates (new in bold) for referenced faunas from southern South America and main references.

Abbreviation	Faunas	Code (Fig. 1)	Latitude	Longitude	Age (mean) Ma	References
AndLo	Andalhuala Fm, lower section	2–4	− 27.20º	− 66.90º	7.14 – 5.64 (6.39)	^39^
AndUp	Andalhuala Fm, upper section	2–4	− 27.20º	− 66.90º	5.64 – 3.66 (4.65)	^39^
CACal	Cerro Azul Fm, Caleufú fauna	8	− 35.70º	− 64.70º	−	^25^
CAChas	Cerro Azul Fm, Chasicoan fauna	21	− 37.40º	− 65.50º	−	^25^
CAHLo	Cerro Azul Fm, lower “Huayquerian" fauna	9–12, 14, 18–20, 22	− 37.18º	− 63.58º	−	^25^
CAHUp	Cerro Azul Fm, upper “Huayquerian" fauna	13, 15–17	− 36.73º	− 64.27º	−	^25^
Chap	Chapadmalalan/Chapadmalal Fm	23	− 38.16º	− 57.62º	**3.74** – **3.04** (3.385)	^20^, this work
Chas	Chasicoan/Arroyo Chasicó Fm	26	− 38.60º	− 63.00º	9.23 ± 0.09	^19^
Jar	Chiquimil Fm, Jarillal member	2, 3	− 27.20º	− 66.90º	8.70 – 7.14 (7.92)	^39^
LTA	Loma de las Tapias Fm, faunal assemblage A	7	− 31.45º	− 68.60º	−	^117^
LTB	Loma de las Tapias Fm, faunal assemblage B	7	− 31.45º	− 68.60º	7.00 ± 0.90	^117^
MH	Montehermosan/Monte Hermoso Fm	29	− 38.97º	− 61.70º	−	^26^
PPint	Palo Pintado Fm	1	− 25.40º	− 65.90º	8.80 –5.27 (7.04)	^118^
QSLo	lower "Irenean", Quequén Salado River	28	− 38.83º	− 60.54º	**5.17 ± 0.08**	^21^, this work
QSUp	upper "Irenean", Quequén Salado River	27	− 38.63º	− 60.61º	**4.33 ± 0.06**	^21^, this work
Sal	Salicas Fm	5	− 28.30º	− 67.00º	ca. 8.00	^119^, F.J. Prevosti pers. obs
SAnd	Sanandresian/San Andrés Fm	24	− 38.18º	− 57.65º	2.60 – 1.78 (2.19)	^6,7,20^
TN	Toro Negro Fm	6	− 28.70º	− 68.30º	6.87 – 4.95 (5.91)	^120,121^
Voro	Vorohuean/Vorohué Fm	25	− 38.18º	− 57.65º	2.90 – 2.60 (2.75)	^6,7,20^

When more than one locality is noted for a fauna, the mean geographic coordinates are presented. When a locality has dates for its upper and lower limit, the midpoint is reported in brackets and is used for regressions. Age (Ma, million years before present) corresponds to radioisotopic dates, with the exception of Voro and SAnd which are based on paleomagnetic studies^20^.

On analysis, one aliquot of a sample from QSLo presented extensive evidence of excess argon in the first half of the spectrum, but the final steps representing about 50% of released ^39^Ar yielded an age of 5.17 ± 0.08 Ma. The large excess in the initial steps suggests that this should be regarded as a maximum age. Two aliquots of a second sample from QSLo yielded an age of 10.5 ± 0.6 Ma as well as a much older age of 18.8 ± 1.6 Ma, but both samples have very low % radiogenic and we consider these results to be unreliable. A single aliquot of a sample from QSUp gave an age of 4.33 ± 0.06 Ma.

The two aliquots of a sample from level VI of the Chapadmalal Fm were measured in two separate irradiations, and provided ages of 3.98 ± 0.19 and 3.74 ± 0.05 Ma. We infer the true age is likely close to the second result (3.74 Ma). A single aliquot of a sample from level X of the Chapadmalal Fm produced a result of 3.04 ± 0.06 Ma. According to our data, the Chapadmalal fauna was deposited over a minimum of ca. 0.7 Ma (Zanclean–Piacenzian), which firmly places it in the late, but not the terminal, Pliocene.

Our results close almost a century of argument concerning the relative antiquity of the “Irenean”^12,21–23^. The controversial lower fauna from the Quequén Salado River can now be shown to be of Zanclean age (5.17 Ma) and thus well within the Early Pliocene. Its upper fauna is also Early Pliocene (4.33 Ma), a point previously unclear. The lower fauna is within the range of error for dated escoria from Cantera Vialidad (5.28 ± 0.04 Ma, Cerro Azul Fm^16^), although few taxa have been recorded from this locality.

### Estimated age of Monte Hermoso and Cerro Azul Fms

To infer the age/order of faunas lacking independent geochronological dating in the Pampean Region, we undertook Maximum Likelihood-Appearance Event Ordination (AEO^24^) in combination with other multivariate methods (see "Material and Methods").

To explore the potential impact of provincialism on AEO, we conducted two analyses (AEO 1, all relevant assemblages included; AEO 2, only assemblages from Pampean Region; Fig. 1). Both AEO1 and 2 results placed the MH fauna chronologically below, and consequently at a position older than, QSUp, but above and therefore younger than QSLo and CACal (but see below). Further, AEO 2 analysis placed the Cerro Azul assemblages^25^ in the following order: CAChas, CAHLo, CAHUp, and CACal (from older to younger); CAChas is above Chas, and CACal below QSLo (Supplementary Table S2, S3).

**Figure 3:**
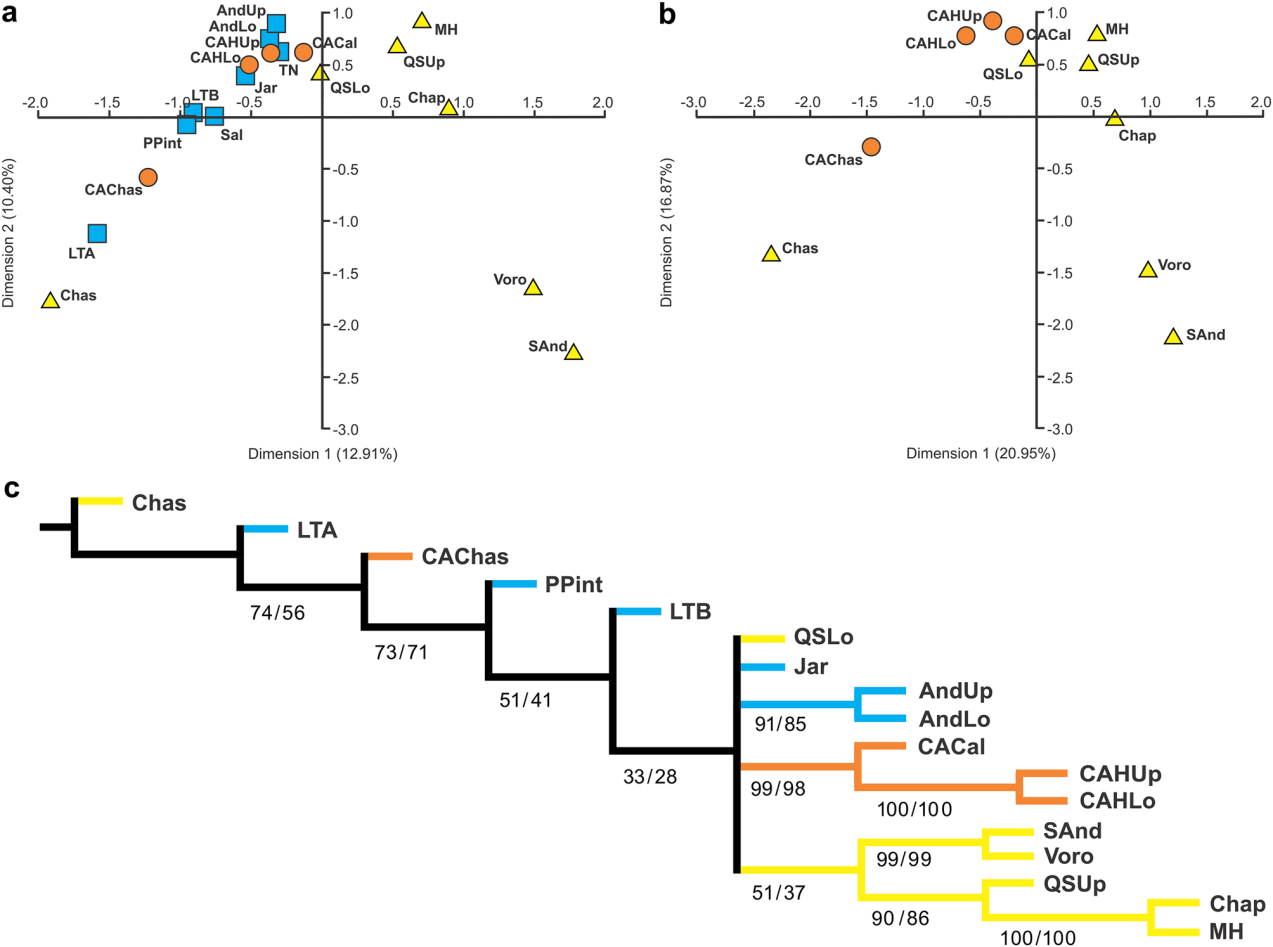
Faunal ordination, similarities and regionalism of the Neogene of Argentina. (**a**) Biplot of the first two axes of Correspondence Analysis (CA) using the complete sample; (**b**) biplot of the first two axes of Correspondence Analysis (CA) using the Pampean Region sample; (**c**) Parsimony Analysis of Endemicity (PAE), reduced strict consensus of four most parsimonious trees of 638 steps, excluding Sal and TN, under “standard” parsimony. Numbers below branches correspond to branch support (frequencies/difference of frequencies, respectively)*.* Yellow triangles (a, b) and lines (c): faunas from Buenos Aires Province; orange circles (a, b) and lines (c): faunas from La Pampa Province (both within Pampean Region); blue squares (a) and lines (c): faunas from Cuyo and Northwestern regions of Argentina. Abbreviations in Table 1. Tree in (c) was obtained with TNT 1.5 (freely available online: http://www.lillo.org.ar/phylogeny/tnt/) and edited with LibreOffice Draw 7.0.3.1 (freely available online: http://www.libreoffice.org/).

Non-Metric Multidimensional Scaling (NMDS), Principal Coordinate Analysis (PCO), and Correspondence Analysis (CA) provided distributions of faunas similar to those with AEO along the first axis, mainly in correlation with known faunal ages (Fig. 3a,b; Supplementary Fig. S4, S5). Sanandresian (SAnd), Voro, Chap, MH, and QSUp appear distantly separated from the other faunas, their order reflecting their relative antiquity, especially in analyses that include only Pampean faunas. In most cases, the position of QSUp in relation to Chap and MH agrees with the AEO results, but other statistics resulted in somewhat different ordinations (Supplementary Fig. S4, S5).

For the Monte Hermoso (MH) fauna, the median age derived from the seven best-performing equations is 4.3 Ma, with median maximum and minimum ages of 4.741 and 3.728 Ma, respectively (Supplementary Table S5). These results support the conclusion that MH is exclusively Early Pliocene (Zanclean), with the median estimated age very close to the ^40^Ar/^39^Ar escoria age of QSUp (4.33 Ma). An Early Pliocene age for the MH fauna is in good agreement with several previous treatments^5–7,10,13,21,22,26,27^, establishing that the Montehermosan did not begin in the Late Miocene (contra refs.^11,28)^.

For the Cerro Azul assemblages, estimated ages cover the gap between Chas (9.23 Ma^19^) and QSLo (5.17 Ma): CAChas: 7.821 (8.925–7.376 Ma); CAHLo: 6.648 (7.239–5.959 Ma); CAHUp: 6.192 (6.935–5.464 Ma); CACal: 5.773 (6.214–5.115 Ma) (Supplementary Table S6). Previously, the outcrops of the Cerro Azul Fm were assigned to either Late Miocene or Pliocene, based on fossil vertebrates from different Pampean localities^29,30^.

### Faunal similarities and regionalism

Parsimony analysis of endemicity^31^ (PAE) was used to explore faunal clustering and the influence of chronology and geography (Fig. 3c; Supplementary Fig. S6a, S6b). Older faunas such as the Loma de las Tapias Fm assemblage are closer to the root (Chas) or form a basal polytomy that includes groups within which more recent faunas are nested. This polytomy includes a few resolved groups: most Buenos Aires Province faunas form a grouping that includes younger faunas (SAnd and Voro), while Pliocene faunas (QSUp, MH, and Chap) form another group. A second “clade” in the polytomy includes the Andalhuala Fm faunas, while a third gathers together the “Huayquerian” faunas of Cerro Azul (CACal, CAHLo, CAHUp), within which CAHLo and CAHUp are the most closely related. The reduced consensus (Fig. 3c), excluding Salicas and Toro Negro faunas, gives better resolution to the base of the tree, resolving CAChas, the Palo Pintado Fm fauna, and the Loma de las Tapias Fm assemblage B as successive sister groups of a polytomic “clade” that includes QSLo, the Jarillal member of the Chiquimil Fm, and the “clades” described above. PAE analysis disallowing reversions results in a very similar tree (Supplementary Fig. S6b).

Multivariate analyses and AEO scores also reveal the influence of geographic distribution: longitude explains ca. 28% of the first multivariate analysis axis and AEO scores, while latitude explains ca. 54% of the second axis using the all-localities grouping (Supplementary Table S7). Limiting attention to the faunas of Buenos Aires and La Pampa provinces, latitude is seen to be significantly correlated only with the first axis of the NMDS based on Corrected Forbes coefficient (62% of explained variation). Longitude is significantly correlated with the first axis of the multivariate analyses and AEO scores, explaining ca. 67% of the variation (Supplementary Table S7).

### The changing pattern of diversity

To explore the diversity and turnover of vertebrate faunas in the Pampean Region we applied different metrics of diversity, speciation, and extinction^32,33^ (see "Material and Methods") using two complementary approaches. Approach 1 includes observed taxa only, while approach 2 includes observed and “Lazarus” taxa^34^. We first analyzed each assemblage from the Pampean Region, and in a second set of analyses we paired some assemblages (e.g., MH + QSUp; see "Material and Methods"). Support for the inferences made in following sections is presented in Fig. 2 and Supplementary Fig. S7–S11.

Taxonomic diversity (richness) is relatively low in CAChas, QSLo, CAHUp, CACal, and QSUp, but attains higher values in CAHLo, MH, and Chap. It decreases again in Voro and SAnd, attaining values similar to Chas. With the inclusion of “Lazarus” taxa, the pattern obtained is similar, but the diversity of CAHUp reaches that of Chas. Faunal pairs produce a very similar pattern with both approaches; CACal + QSLo display a drop in diversity. Net diversification is higher in Chas and CAHLo, followed by MH and Voro, and is low in CAHUp, Chap, and SAnd (other faunas have intermediate values). Once again, faunal pairing gives a very similar pattern in both approaches. The pattern for turnover rates is likewise similar to that for net diversification for both approaches, although Chap displays a drop.

Origination rates are high for Chas, CAHLo, MH, Chap, and Voro, low for SAnd, and very low for QSUp in both approaches. Faunal pair analyses show similar results, but MH + QSUp, Chap, and Voro have a lower origination rate than Chas and QSHLo + QSUp. Approaches 1 and 2 are consistent in identifying Chap as having the highest extinction rates in the analyses of single and grouped faunas. For other faunas, extinction rates vary. Subsampling analyses provide similar results, with some exceptions (e.g., higher origination rate for Voro).

### Mammalian community structure during the Late Miocene–Pliocene

**Figure 4:**
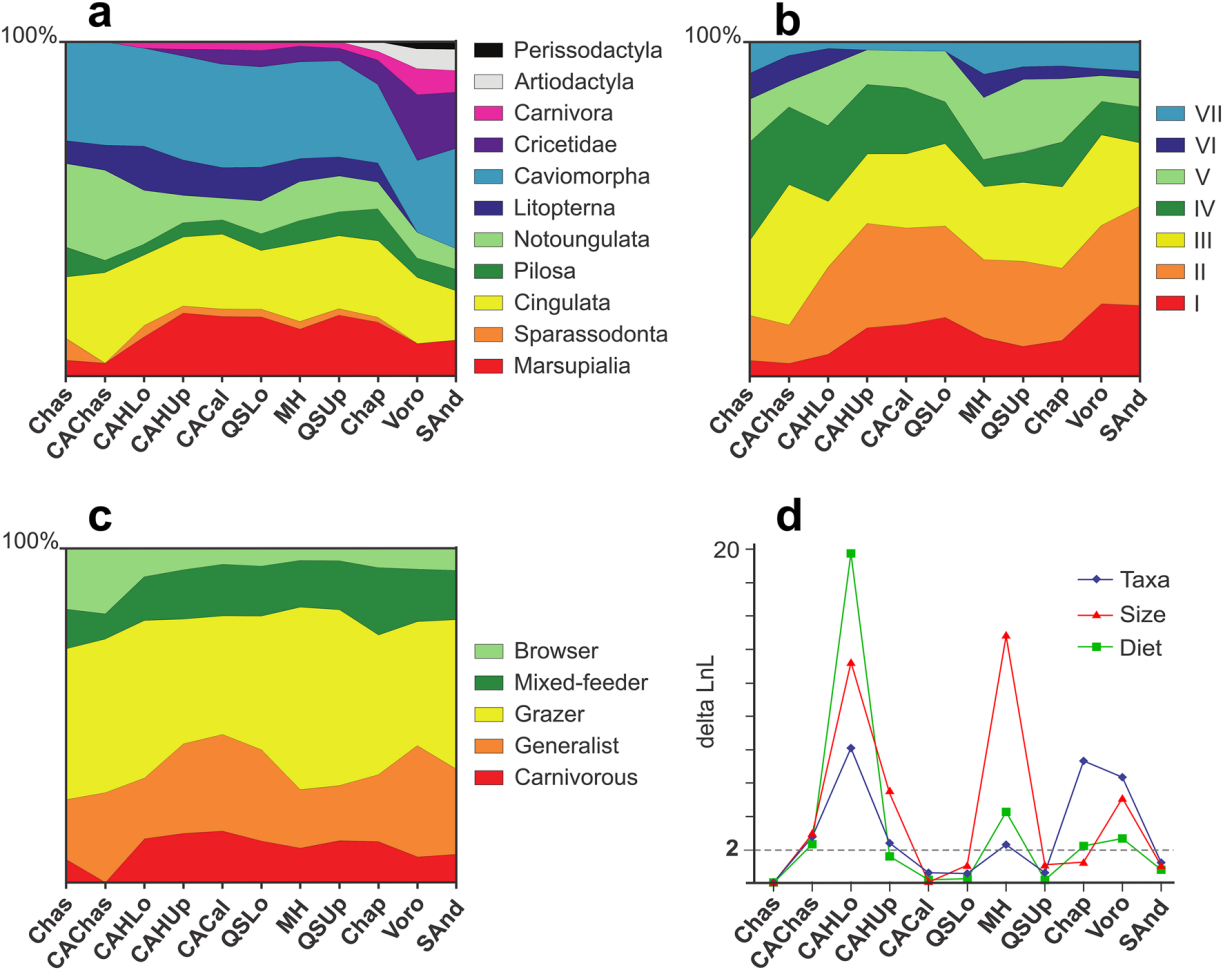
Change in structural parameters of investigated faunas through time (approach 2). (**a**) Proportion of taxonomic groups across the Neogene faunas; (**b**) changes in proportion of body size classes (I, less than 100 g; II, between 100 g and 1 kg; III, 1–10 kg; IV, 10–100 kg; V, 100–500 kg; VI, 500–1000 kg; and VII, more than 1000 kg); (**c**) proportion of dietary classes across the Neogene faunas; (**d**) Log-Likelihood difference between successive faunas (significant cutoff [2] is indicated with a dashed line). Abbreviations in Table 1.

Mammalian taxonomic composition and trophic structure (in terms of diet and body size) were explored through time using the multinomial likelihood statistic^35^, and the same two approaches (i.e., excluding vs. including “Lazarus” taxa; Fig. 4 and Supplementary Fig. S11–S13). Except for CAHUp, QSUp, and SAnd in approach 1, the changes in proportions of taxonomic groups within the faunal analyses are significant in most assemblages during the Late Miocene–Pliocene. However, major modifications occur in CAHLo and MH, while Chap and Voro show more moderate differences. In approach 2, observed changes are similar, but, in this case, MH displays moderate change, while change is more exaggerated in Chap and Voro. A reduction in notoungulate diversity is notable from the time of CAHLo onward. CAHLo also witnesses the first appearance of carnivorans (procyonids), while cricetid rodents first appear in the temporally subsequent faunas, CAHUp and CACal. Sparassodonts collapse in diversity after Chas and become extinct in Chap, but crown group marsupials are more diverse through CAHLo–Chap times. First appearances of artiodactyls (tayassuids) and perissodactyls (equids) occur in Chap and Voro, respectively. Diversity is reduced in litopterns, but increased in carnivorans and cricetids after Chap time. Analyses of single and paired faunas present a similar pattern of change in taxonomic diversity, although in approach 1 only CACal + QSLo, MH + QSUp, Chap, and Voro are significant. Also in approach 1, MH + QSUp is the only group exhibiting drastic change. In approach 2, change in MH + QSUp is not significant, while it is much more apparent in Chap and Voro.

Except in the case of CACal and SAnd, approach 1 reveals statistically significant changes in the distribution of body size classes in most faunas, with MH and Chas showing the greatest degree of change. In contrast, approach 2 shows significant changes in body size distribution in CAHLo and MH, but not in QSLo, QSUp, or Chap. Overall, the two approaches agree in showing an increase in frequency of the small body size classes I and II after Chas or CAChas time, respectively, with maximum values achieved in our samples of Voro and SAnd (Class I) and CAHLo (Class II). Class IV undergoes reduction after CACal time, while CAHUp, CACal, and QSLo faunas either lack or have a very low proportion of the larger body size classes VI and VII. Grouped faunas present a similar pattern of change in body mass distributions.

With both approaches, changes in the proportions of dietary classes only reach significance in the case of CAChas, CAHLo, and MH, with the last two faunas showing the most marked change. In approach 2 only, changes in Chap and Voro are also significant. Both approaches show a decrease in browsers and an increase in carnivorous taxa after Chas and CAChas time, in Voro and SAnd. Mix-feeding herbivorous mammals become more frequent after Chas and CAChas time. Analyses of grouped faunas follow a similar trend except in the case of Voro and SAnd in approach 1, and CACal + QSUp and SAnd in approach 2.

## Discussion

### Understanding GABI: an improved chronology

Our new ^40^Ar/^39^Ar ages establish, for the first time, a sound radioisotopic basis for bounding the ages of several traditionally-recognized Neogene faunas of the Pampean Region of South America. The statistically-based biochronological analyses presented here also help to better position other faunas not yet securely dated and avoids a circular argument and subjectively recognized “evolutionary grades” as a basis for age assignments^36^, contra ref.^22^. The new data have a bearing on dating the first part of the GABI, as well as calibrating the extinction of certain autochthonous clades. Several localities of the lower “Huayquerian” of the Cerro Azul Fm (i.e., Quehué, Telén, Salinas Grandes de Hidalgo^5,37^; Fig. 1; Table 1), with a median age estimated at 6.648 Ma (range 7.239–5.959 Ma), have yielded fossils of the procyonid carnivoran *Cyonasua* spp. This is now the oldest confirmed record of carnivorans in the Pampean Region and overlaps the oldest known South American record for Procyonidae from El Jarillal Member of the Chiquimil Fm in Catamarca Province (range 7.31–7.14 Ma based on a capping dated toba, paleomagnetism, and sedimentary rates^38–40^; Fig. 1, Table 1). These are currently the oldest known records of a Cenozoic North American mammal immigrating to South America (but see^41,42^ and^43^ for discussions). Remains assigned to *Cyonasua* sp. from Perú^44,45^ also overlap this FAD^46^.

Another important result is the confirmation of the Late Miocene occurrence of cricetid rodents (Sigmodontinae) in South America, a persistently controversial topic^21,36,47–49^. Cricetid remains^50^ claimed to have come from the lower “Irenean” of the Quequén Salado River are now lost or never existed. The only other pertinent discovery concerns a tiny molar fragment from the Late Miocene Andalhuala Fm (ca. 7 Ma) that was originally assigned to Sigmodontinae^51^, but later questioned due to its fragmentary condition^52^. The Cerro Azul assemblages from Caleufú and El Guanaco localities include cricetid remains^36–47^. By combining our new radioisotopic dates with biochronology (Supplementary Table S6), a further refinement of the sigmodontine temporal record is now possible. Our results indicate a median age of 5.773 Ma (range 6.214 –5.115 Ma) for the Caleufú assemblage (CACal) and 6.192 Ma (range 6.935–5.464 Ma) for that of the upper “Huayquerian” (CAHUp). Confirming Reig’s seminal hypotheses^53^ these estimates now stand as the oldest age-confirmable FADs for this highly speciose group of neotropical rodents^36,47,54^. Although these datings represent an extension of the biochron of the group in South America, we are still far from the dates derived from molecular-based phylogenies that predict its presence during the Miocene, with ages ranging from 20 to 9 Ma^55–57^. Many of the estimates based on molecular clocks use few or questionable fossils (e.g., *Prosigmodon*) to calibrate the main nodes ^55–57^. The gap that exists between fossils and predictions through molecular studies also implies that the ancient evolutionary history of sigmodontines took place in the northern portion of South America. The recent record of Pliocene cricetids in Venezuelan deposits^58^ represents a promising finding that introduces fresh data for this topic.

The single tayassuid specimen from level III of the Chapadmalal Fm^59^, which lies just below the dated level VI (ca. 3.74 Ma), helps to further constrain the FAD for this artiodactyl family in South America. Tayassuids from putative Late Miocene levels in Peru have been previously reported^42^, but as their geographic and stratigraphic context is imprecise, their age cannot be corroborated at present^43,60^.

The oldest record of Camelidae in South America, also from the Chapadmalal area^61^, consists of a fragmentary skull of *Hemiauchenia* sp. This fossil was originally assigned to the Barrancalobian^6,7^, but later to the Chapadmalalan^62^. More recently, fossils attributable to the camelid *Hemiauchenia* have been recovered from Calera Avellaneda, central Buenos Aires Province, from levels of putative Chapadmalalan Age as indicated by faunal associations and paleomagnetic dating (Gauss chron, 3.55–2.59 Ma^61^). Although the Calera Avellaneda locality has not been properly constrained radiometrically^63^, the fossils may be of roughly the same age as a camelid tooth from northern Colombia dated by ^87^Sr/^86^Sr and mollusk biostratigraphy to the Late Pliocene (ca. 3.2 Ma, range 3.4–2.78 Ma^64^). However, these dates extend over a large interval (5.71–1.57 Ma)^65^ and have a mean error of ca. 1.13 Ma (calculated from^65^) which does not exclude an age younger than Chapadmalalan. The oldest records for other Holarctic families, including canids, mustelids, and equids, are also from the Chapadmalal area (Vorohué Fm; ca. 2.9–2.6 Ma^1,6–8,27^).

In the case of South American terror birds (Phorusrhacidae), the latest ages for large and medium-sized taxa from upper levels of the Chapadmalal Fm can now be tightly constrained to ca. 3.04 Ma (Fig. 2). Fragmentary remains of smaller terror birds (< 10 kg) are known from the Late Pleistocene of Uruguay^66^, which could imply that they persisted into the last part of the Quaternary. Terror birds survived in North America until the early Pleistocene^67^. Several lineages of metatherians also underwent marked turnover during the late Neogene. The last sparassodonts disappeared at the same time as large terror birds. The last extra-Andean record for shrew opossums (caenolestids) is Early Pliocene (5.17 Ma; QSLo)^68^, while the last known argyrolagids (small ricochetal marsupials possibly related to caenolestids) occur during the Late Pliocene–Early Pleistocene (Vorohué and San Andrés Fms; 2.9–1.78 Ma), about the time of the last sparassocynins (carnivorous opossum-like marsupials) and large, likely carnivorous didelphids (e.g., *Thylophorops*)^27,69–72^.

### Faunal evolution, environmental changes, and biotic interaction

During the Late Miocene–Early Pliocene, South American vertebrate faunas underwent massive transformations (Figs. 2, 3, 4, Supplementary Fig. S7–S13), beginning with the Arroyo Chasicó fauna which only shares four taxa with older faunas (including the youngest examples of primitive peltephilid armadillos^14^). The extinction rate was high in the Arroyo Chasicó and Chapadmalal faunas^73^, causing a sharp drop in the net diversification rate of the latter. Associated faunal changes include the complete disappearance of sparassodonts and most terror birds, a decrease in the diversity of ground sloths and litopterns, an increase of cricetid, artiodactyl, and carnivoran taxa (including the first records of canids and mustelids in South America), and the first appearance of equids^6–8,13,27,74^ in the Vorohué Fm.

Discussions concerning the factors that might have triggered faunal turnovers of this magnitude in South America have classically focused on either biological interactions or environmental changes, termed the Red Queen and Court Jester hypotheses, respectively, in the recent literature^75,76^. The Red Queen hypothesis focuses on the effects of competition between native lineages (Simpson’s strata 1 and 2) and immigrants (stratum 3) during the GABI, as in the classic example of native metatherian predators vs. immigrant placental carnivorans^73,77^. However, recent reviews have not supported the idea that these new arrivals directly caused the extinction of autochthonous predator lineages, or that competitive displacement occurred among different metatherian clades^5,27,78^ (Fig. 2).

In the Court Jester hypothesis, the rise of the Andes and the gradual desertification of the continent along the east side of the southern cone are considered to be the chief causes of faunal transformation since Middle Miocene times^14,27,73,78^. The Central Andes experienced uplift during the late Early Miocene^79^ (Fig. 2), and the Bolivian Altiplano underwent further significant uplift around 6–5 Ma, when it almost achieved its present altitude (ca. 4000 m.a.s.l.^80^). Further contributing to this process were variations in atmospheric circulation and global climatic change^81,82^. The Late Miocene (ca. 6–5.4 Ma) and mid-Pliocene (3.5–3.3 Ma) cooling events caused aridification in northwestern Argentina and triggered a shift to a C4-enriched ecosystem^72–74^. This led in turn to an increase in C4 plants in mammalian diets^81–83^ and probably an increase in hypsodonty in several mammalian lineages^84^, as well as facilitating the spread of xeric shrublands^85^. In central Argentina about this time (Arroyo Chasicó assemblage, ca. 9.23 Ma; lower “Huayquerian” of Cerro Azul Fm, ca. 6.648 Ma), herbivore diets would have been largely dominated by C3 plants, but isotopic analyses indicate that a number of species were already relying on mixed diets of C3-C4 plants during the Early Pliocene (Monte Hermoso Fm, ca. 4.30 Ma). This corresponds to the establishment of more open biomes and expansion of C4 grasses^5^.

Other geographical factors (e.g., precipitation decrease in western part of southern cone) probably had a profound impact on mammalian faunas in the period after 7 Ma. Isotopic data (^18^O, ^13^C) suggest that the environments in which the Monte Hermoso fauna lived were affected by Pliocene global warming^5^. The occurrence of termite nests during this interval^86^ and results of a phytolith study^87^ support the idea that a climate warmer than at present existed during the deposition of the Chapadmalal Fm, with vegetation dominated by palms and C4 grasses, and other components forming bushy savannas^87^. After 3.3 Ma cooling accelerated, marked by the great expansion of continental ice sheets in the northern hemisphere^88,89^. Glaciations occurred episodically in Patagonia from the end-Miocene onward (7.38–5.05 Ma, 4.8–4.7 Ma, 3.68–3.55, ca. 3.46, ca. 3.20, and > 2.79 Ma), and were more frequent thereafter in the Pleistocene^90^.

Climatic changes on these scales surely affected the vegetation and vertebrate faunas, and were probably the most important factor in driving faunal turnovers, extinction, and origination events, reduction of browsers and increase in grazers after Chasicoan time, and still other transformations affecting body size and diet of individual taxa. For example, terminal Chapadmalalan extinctions were coincident with a sharp drop in global temperature related to the establishment of the so-called icehouse climate regime^13,74^. Although it is difficult to compare faunal events in South America with those that occurred elsewhere, because of differences in time binning, taxonomic scale analysis, and/or methodology, our results are in agreement with numerous studies showing serious impacts on faunas worldwide during the late Neogene and early Quaternary^74,91–94^.

A less examined potential cause of faunal turnover and extinction is meteorite collisions^1^. If all of our sampled and dated escorias are the result of extraterrestrial impacts, as seems quite plausible^16^, then there were evidently several Neogene events that could be correlated with faunal changes. Impacts occurred at 9.23 (Arroyo Chasicó assemblage), 5.28 (Cantera Vialidad, Cerro Azul Fm), 5.17 (lower fauna of the Quequén Salado River), 4.33 (upper fauna of the Quequén Salado River), 3.74 and 3.04 Ma (Chapadmalal Fm) (Fig. 2). Only two craters have been identified in the region^17^, which implies that the meteorites usually disintegrated in the atmosphere. In addition to the correlations just noted, at least other four “escoria events” occurred during the Middle Pleistocene–Holocene^16^. Together, these records imply an impressive series of impacts during the South American late Neogene–Quaternary, but the question of interest is whether they had any acute or long-lasting effect on the biota. Here, the evidence is far from clear. By comparison with the most consequential impact event during recent Earth history—Chicxulub at 66.04 Ma^95^—the Pampean record is less impressive.

Although it is obviously difficult to assess whether such collisions might have played an independent role in forcing extinctions in Neogene South American faunas, in principle there should be a correlation between catastrophic effects that can be plausibly linked to a given event, such as a meteoritic impact and coeval organismic loss, as proposed by the “sloshing bucket” model of evolution^96^. Application of this idea to the study area will require much additional information about escorias, impact timing, and closely-spaced downstream effects of extraterrestrial visitors.

## Conclusions

The new ^40^Ar/^39^Ar dates for supposed impact-related escorias from the Argentinean Atlantic coast, together with the statistical biochronological analyses developed here, improve various aspects of the Age/Stage framework for Neogene South American faunas based on the fossil record of the Pampean Region. These results refine the FAD for several immigrant lineages (e.g., cricetids in Late Miocene, tayassuids before 3.74 Ma) and last occurrences (extinction) of some major autochthonous clades in South America (sparassodonts and most terror birds, disappearing ca. 3 Ma.). At present, faunal turnovers in the late Neogene are plausibly interpreted as knock-on effects of global climatic changes. In particular, although meteoric impactors cannot be excluded as a relevant factor, the Late Pliocene extinction event is most probably related to the initiation of the icehouse climate regime that has dominated the last three million years of Earth history.

## Materials and methods

### Localities and chronological framework

Escorias were recovered for the purpose of isotopic dating from several localities/units within the Neogene sequence of Buenos Aires Province: Paso del Halcón and Cascada Grande (“Irenean”^21,22^), Playa Las Palomas and Playa La Estafeta (Chapadmalal Fm^97^, as well as the Punta San Carlos and Playa de Los Lobos Alloformations^98,99;^ Fig. 1, Table 1). We follow the Late Miocene–Quaternary South American Age/Stage sequence as defined by Cione and Tonni^6,7^ with relevant modifications^8,11^.

### Chronometric dating: methods

Using ^40^Ar/^39^Ar, we dated escorias from four distinct levels of two Neogene formations in the Pampean Region^100^. Bulk samples of escorias were gently crushed and the clearest glass fragments were hand-picked under a binocular microscope. Several milligrams of glass fragments were loaded into pits (wells) in aluminum irradiation disks. Each disk contains 12 pits, two of which were loaded with Fish Canyon sanidine monitor. The packages were irradiated for 8 h with cadmium shielding and rotation at the United States Geological Survey TRIGA reactor (Denver, Colorado). Samples were loaded into tantalum tubes and evacuated to ultrahigh vacuum in a chamber with a quartz glass window. A diode laser was used to incrementally heat the samples. Analyses of monitors and unknowns were corrected for backgrounds and mass discrimination using frequent measurements of blanks and air pipettes. Corrections for nuclear interferences were based on data from previous studies^101^. Calculations for J were based on the estimated age of 28.201 ± 0.046 Ma for Fish Canyon^102^ with decay constants^103^. Isochron results, together with the step-heating spectra for the estimated atmospheric initial from the isochron intercept, are presented in the Supplementary Fig. S1–S3. In all cases the preferred age interpretation is the plateau from the step heating calculation with trapped initial composition estimated from the isochron.

### Interpretation of vertebrate assemblages

Pampean faunal assemblages evaluated in this contribution (Fig. 1, Supplementary Dataset S1) were recorded from the following contexts (in order, west to east): Chas, MH, QSLo, including samples recovered at El Paso, Paso de la Tufa, Paso del Halcón, Paso del Médano, and Usina Vieja localities; QSUp, including samples recovered from Cascada Grande, Cascada del Pampaterio, Molino de la Rosa, Cascada de la Ruta, and Cascada Escondida localities; Chap, Voro, and SAnd. We included also well-sampled, but undated, faunas from the CAChas, CAHLo, and CAHUp. We analyzed the CACal assemblage as a separate entity^25^ in order to assess the current controversy about its age^36,47^.

Voro and SAnd lack detailed chronometric control, but their ages are based on paleomagnetic correlation^20^. We included their faunal assemblages in our analyses because they stratigraphically overlie Chap^20,97^ and are relevant to understanding faunal succession during the Late Pliocene. MH lacks both chronometric and paleomagnetic dating, but its mammalian assemblage constitutes the faunal foundation for the Montehermosan Age/Stage. Its age was explored here using different methods (see below). The Cantera Vialidad assemblage (Buenos Aires Province) was dated to 5.28 ± 0.04 Ma^16^, but it was excluded from our integrative analysis because of its poor fossil content, which consists of only three taxa (i.e., *Chorobates villosissimus*, *Paedotherium* sp*.*, and *Xenodontomys ellipticus*^104^). Exclusion, in this case, does not represent a significant loss for the taxonomic database, because *Paedotherium* sp*.* and *X. ellipticus* were also registered in the more diverse QSLo fauna^21,22^, and both localities arguably represent the same faunal “unit”. Barrancalobian was not recognized as a distinguishable Subage/Substage or fauna^62^.

In order to constrain the age of the Monte Hermoso and Cerro Azul assemblages, and thereby increase our comparative dataset, eight additional vertebrate associations from the Cuyo and northwestern regions of Argentina were analyzed (Fig. 1, Table 1).

The main sources of paleontological data utilized here come from a variety of previous compilations^6,12–14,97^, as well as from contributions summarizing relevant locality and clade information^8,21,22,26,27,74,104^ (Supplementary Dataset S1–S2). The final database included 393 vertebrate taxa from 19 paleontological localities or settings (Table 1), and was used to evaluate the temporal ordination of faunas and evidence of major changes in taxonomic diversity and ecological adaptation during the late Neogene/early Quaternary in the Pampean Region.

### Faunal temporal ordination and age inference

Maximum Likelihood Appearance Event Ordination^24^ (AEO) was employed to place the MH vertebrate association within the sequence of faunas analyzed here. Two runs were conducted, one exclusively using faunas from the Pampean Region and the other incorporating all relevant assemblages.

AEO was performed with PAST v. 2.17c^105^; singletons were excluded and assemblages from the same stratigraphic formation were ordered in sections, with 100 bootstrap replicates. Faunal ordination was also explored via multivariate methods, including Non-Metric Multidimensional Scaling (NMDS), Principal Coordinate Analysis (PCO), and Correspondence Analysis (CA^106^) in PAST v. 4.03^105^. NMDS and PCO were performed Corrected Forbes coefficient^107^ and Bray–Curtis coefficient^108^ distance matrices. Parsimony analysis of endemicity^31^ (PAE) was used to explore faunal clustering and the influence of chronology and geography. Generated trees were rooted with Chas mammal assemblage, and analyses were conducted under maximum parsimony and reversion dismissal (i.e., only taxa that exhibit shared presence can support “clades”). Searches were performed with 1000 random sequence additions plus TBR swapping, and node support was estimated with Symmetrical Resampling with 1000 permutations. All analyses were also explored with TNT 1.5^109^ using the complete database (i.e., when two or more species were identified, the taxonomic category of the genus was included as a separate entry).

AEO and the first two axis scores of the multivariate analyses were regressed to the known ages of faunal assemblages to infer the age of MH and the Cerro Azul Assemblages, using Quantile Regression^110^. Prediction Percentage Error Eqs.^111^ below 10% were used to estimate the age of this undated fauna. Regressions were performed with R version 3.6.1^112^. This permits the age of assemblages lacking radiometric ages to be estimated quantitatively; these hypotheses can be tested in the future with more evidence.

The potential existence of a geographic pattern in the ordination of the faunas in the multivariate analyses was explored in R version 3.6.1^112,^ using Spearman correlation indices and focusing on scores and geographic coordinates for each fauna.

### Faunistic change in the associations from Pampean Region

To explore the diversity and turnover of vertebrate faunas in the Pampean Region, we applied the metrics of diversity, speciation, and extinction^32,33^ using the R package divDyn^113^. Net diversification and turnover rate were calculated following Foote’s eqs.^32,35^. To permit calculation of at least some of these metrics for the Chas and SAnd faunas, we concatenated older (Late Miocene) and younger (Quaternary-Recent) assemblages in two entries. Taxa shared between older faunas and Chas-SAnd were coded as present. Similarly, taxa shared between younger assemblages and Chas-SAnd faunas were coded as present. For the entire set of Pampean Region faunas, two groups of analyses were run. The first group treated faunas as separate units, ordered using available dates and AEO. The second group paired up faunas having similar estimated ages and faunal overlaps, including MH + QSUp, CACal + QSLo, CAHLo + CAHUp, and Chas + CAChas. This last analysis explores the possibility that several of the Cerro Azul assemblages belong to the same fauna based on literature sources^21,22,114^. To control the presence/absence of taxa in different faunas, we performed subsampling analyses using Classical Rarefaction Analysis^115^, with subsampling set at 40% and 1000 iterations using the R package divDyn^113^.

Mammalian taxonomic composition and trophic structure (in terms of diet and body size) through time were explored using the multinomial likelihood statistic^114^. The following taxonomic groups were analyzed: Sparassodonta, Marsupialia, Cingulata, Pilosa, Notoungulata, Litopterna, Caviomorpha, Cricetidae, Perissodactyla, Artiodactyla, and Carnivora. Each taxon was assigned to dietary and body size categories according to published paleoecological studies or anatomical/geometric similarities to living and/or close relatives (see Supplementary Dataset S2). Diets were broadly defined as carnivorous (predators), generalist (“omnivorous”), or herbivorous, with taxa in the last group subdivided into grazer, mixed feeder, and browser subgroups^27,116^. This fairly coarse classification was preferred because information sufficient for finer groupings was not available for all analyzed taxa. Body sizes were grouped as follows: category I, less than 100 g; II, between 100 g and 1 kg; III, 1–10 kg; IV, 10–100 kg; V, 100–500 kg; VI, 500–1000 kg; and VII, more than 1000 kg.

Two types of analyses were performed. The first treated faunas as separate units, ordered using available dates and AEO. The second through paired up faunas having similar estimated ages and faunal overlaps, including MH + QSUp, CACal + QSLo, CAHLo + CAHUp, and Chas + CAChas. Furthermore, we used two different approaches to data grouping for each type of analysis. Approach 1 employed observed taxa only. Approach 2 additionally included “Lazarus” taxa (i.e., range-through taxa, where a taxon absent in one level is present in previous and later levels^34^).

## Supplementary Information

Supplementary Information 1.

Supplementary Information 2.

Supplementary Information 3.
