# Supplementary material for: New radiometric 40Ar–39Ar dates and faunistic analyses refine evolutionary dynamics of Neogene vertebrate assemblages in southern South America
Source: Sci Rep. 2021 May 10;11:9830. doi: 10.1038/s41598-021-89135-1 (PMC8110973; doi:10.1038/s41598-021-89135-1)
Supplement: Supplementary file 1 — Supplementary Information 1. [file 41598_2021_89135_MOESM1_ESM.pdf]

Supplementary Figures and Tables for:

New radioisotopic $^{40}Ar/^{39}Ar$ dates and faunistic analyses refine evolutionary dynamics of Neogene vertebrate assemblages in southern South America

Francisco J. Prevosti, Cristo O. Romano, Analía M. Forasiepi, Sidney Hemming, Ricardo Bonini, Adriana M. Candela, Esperanza Cerdeño, M. Carolina Madozzo Jaén, Pablo Ortiz, François Pujos, Luciano Rasia, Gabriela I. Schmidt, Matías Taglioretti, Ross D. E. MacPhee, and Ulyses F.J. Pardiñas

Corresponding author: Francisco J. Prevosti, Email: protocyon@homtail.com

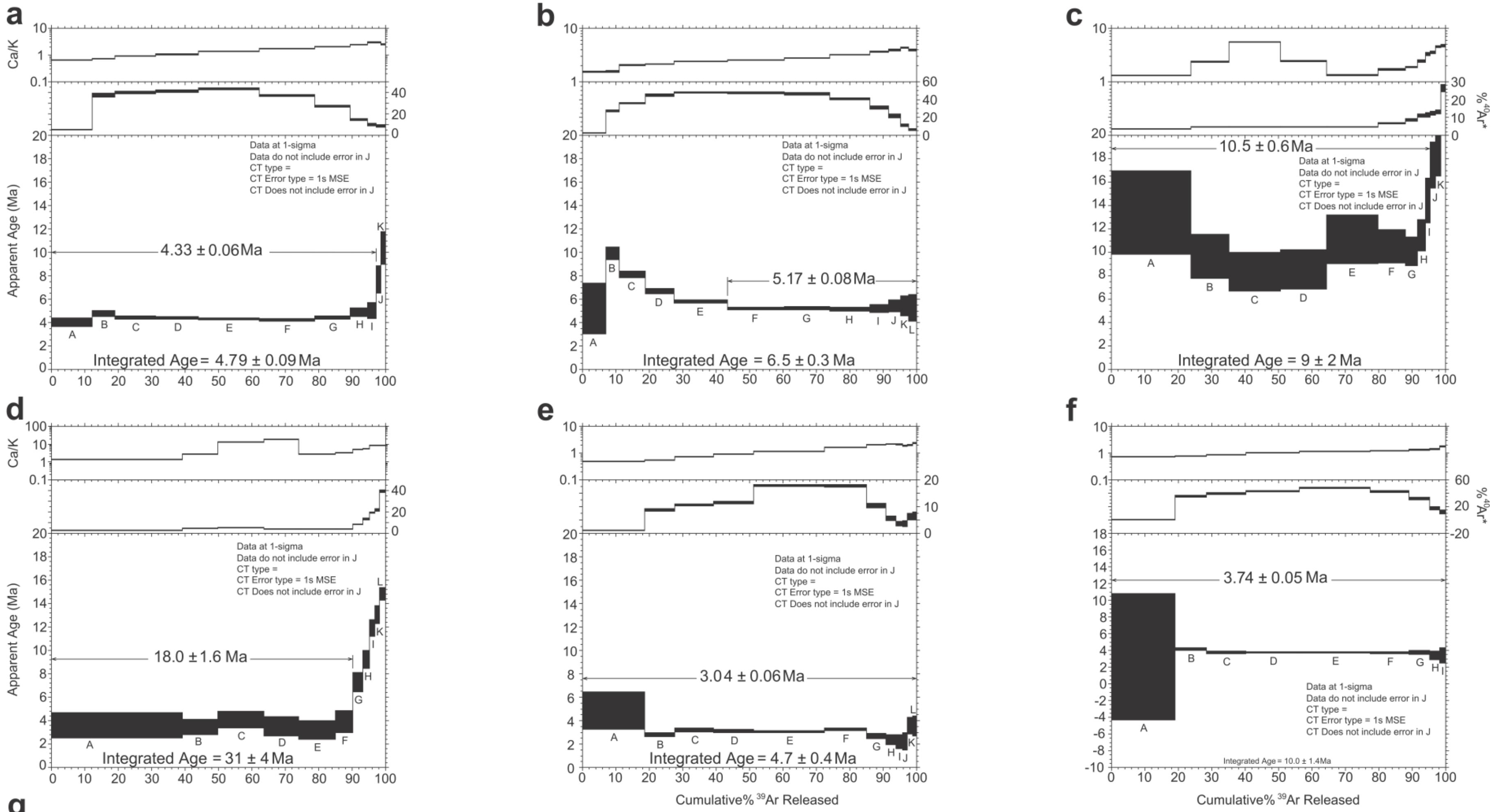

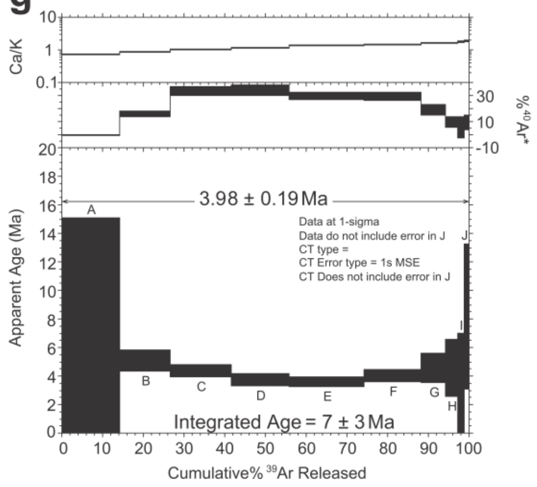

Supplementary Fig. S1. Plots of step-heating 40Ar/39Ar apparent age spectrum (trapped initial) for collected samples. **(a)** 1# Cascada Grande ("Irenean", unit 3); **(b)** 2# Quequén Salado ("Irenean", unit 2); **(c)** 1# Paso del Halcón ("Irenean", unit 2); **(d)** 2# Paso del Halcón ("Irenean", unit 2); **(e)** 1# Chapadmalal Fm (Chapadmalal Fm, level X); **(f)** 1# APSL P3 (Chapadmalal Fm, level VI); **(g)** 2# APSL P3 (Chapadmalal Fm, level VI). Extended data for samples in Supplementary Table S1.

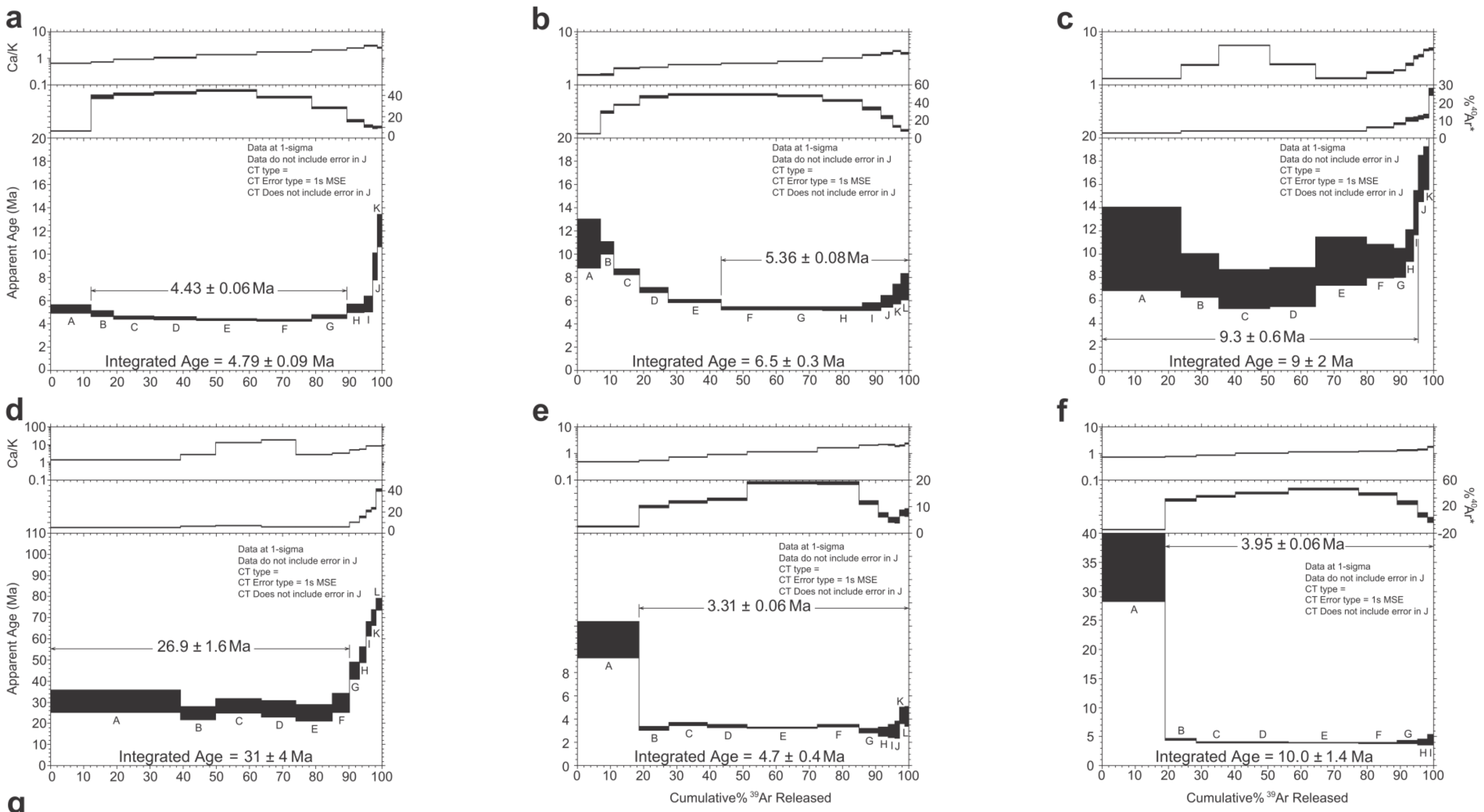

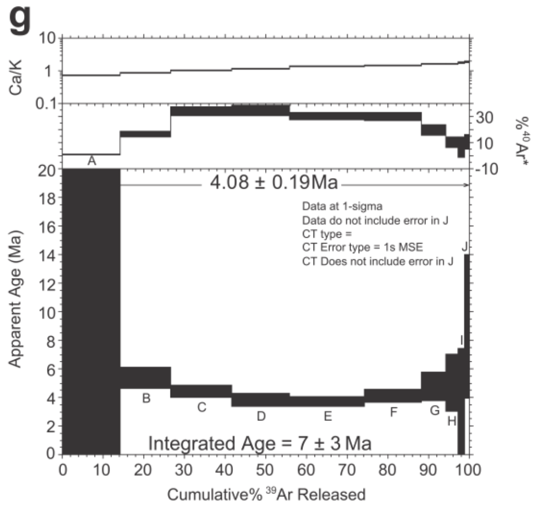

Supplementary Fig. S2. Plots of step-heating 40Ar/39Ar apparent age spectrum (ATM initial) for collected samples. **(a)** 1# Cascada Grande (“Irenean”, unit 3); **(b)** 2# Quequén Salado (“Irenean”, unit 2); **(c)** 1# Paso del Halcón (“Irenean”, unit 2); **(d)** 2# Paso del Halcón (“Irenean”, unit 2); **(e)** 1# Chapadmalal Fm (Chapadmalal Fm, level X); **(f)** 1# APSL P3 (Chapadmalal Fm, level VI); **(g)** 2# APSL P3 (Chapadmalal Fm, level VI). Extended data for samples in Supplementary Table S1.

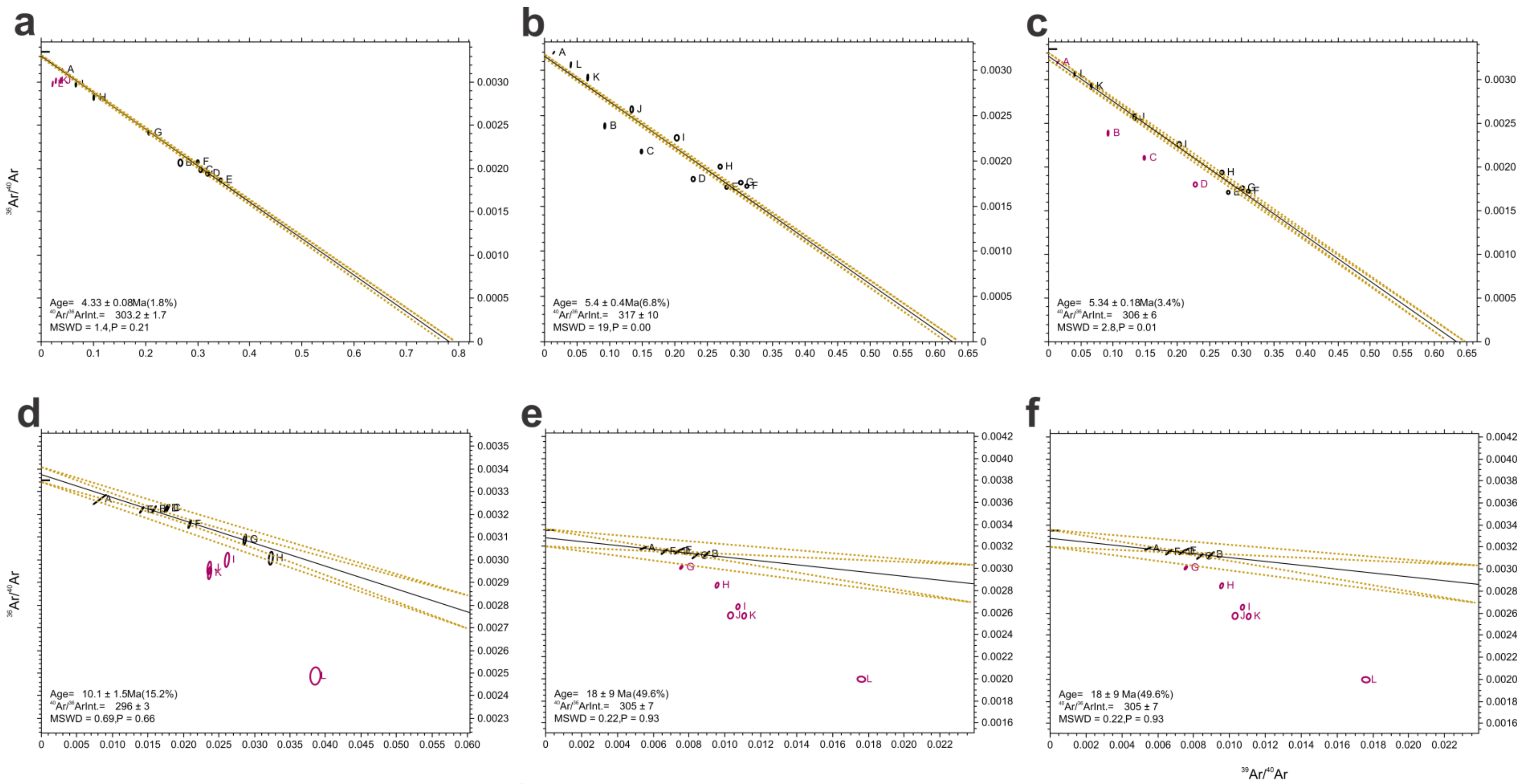

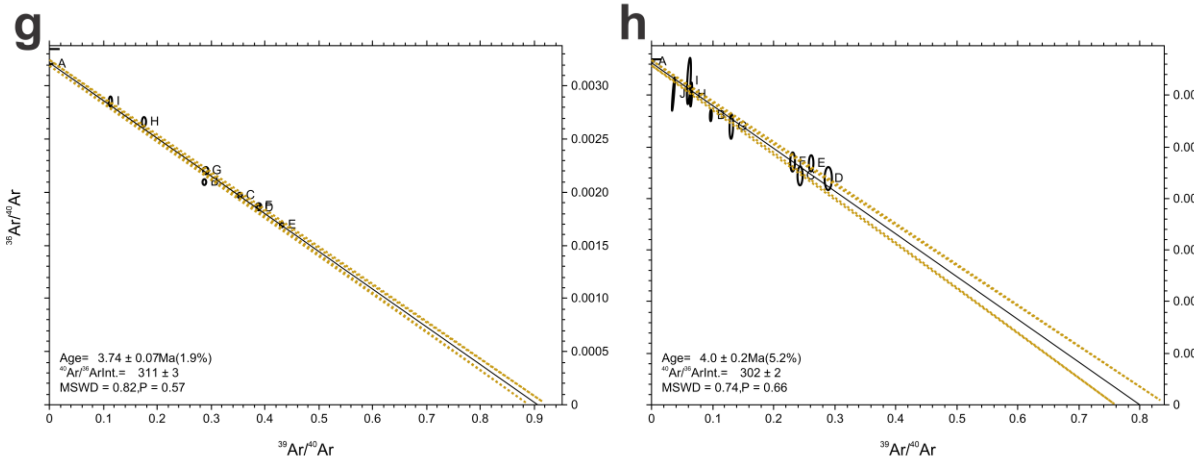

Supplementary Fig. S3. Isochron graph for collected samples. (a) 1# Cascada Grande (“Irenean”, unit 3); **(b-c)** 2# Quequén Salado (“Irenean”, unit 2); **(d)** 1# Paso del Halcón (“Irenean”, unit 2); **(e)** 2# Paso del Halcón (“Irenean”, unit 2); **(f)** 1# Chapadmalal Fm (Chapadmalal Fm, level X); **(g)** 1# APSL P3 (Chapadmalal Fm, level VI); **(h)** 2# APSL P3 (Chapadmalal Fm, level VI). Data in pink omitted from regression. Extended data for the samples in Supplementary Table S1.

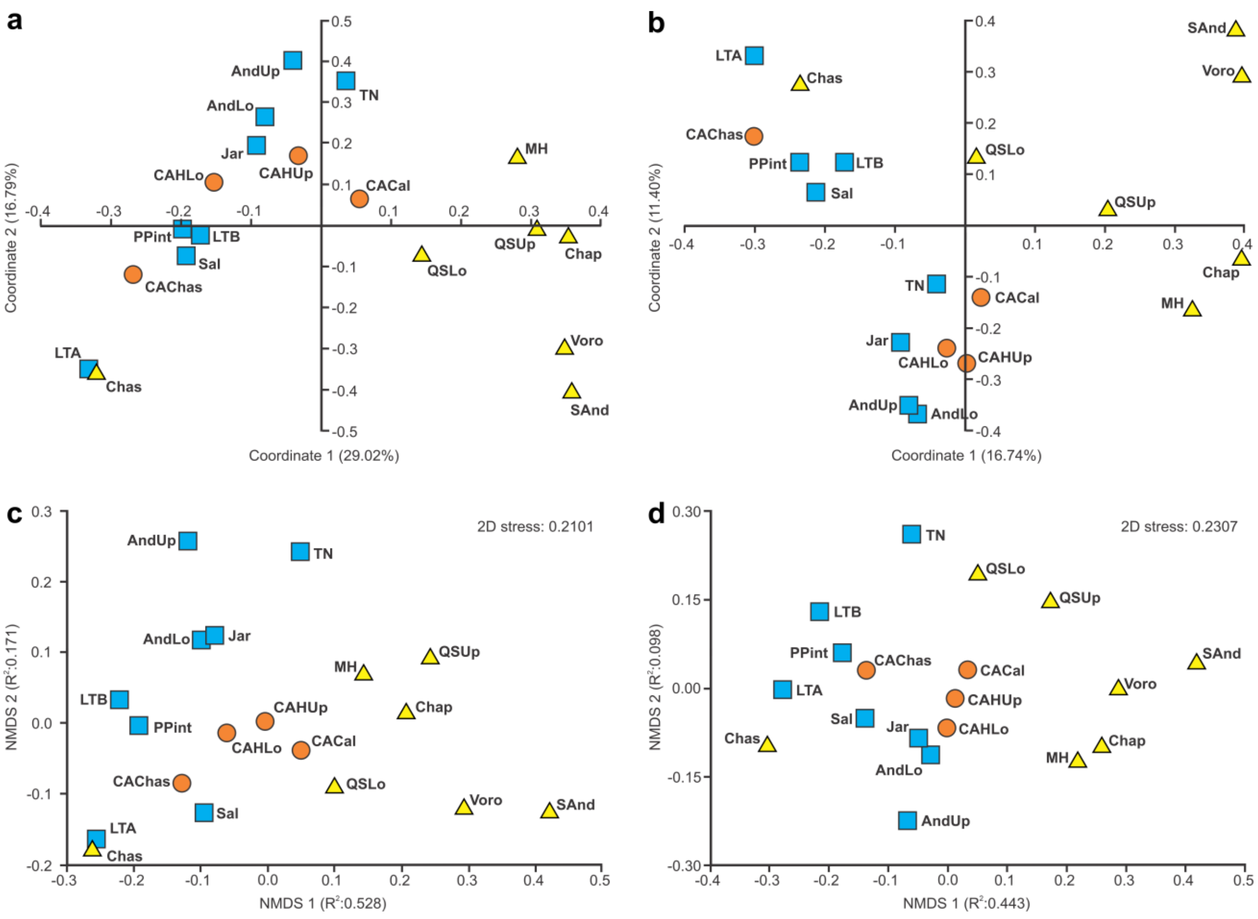

Supplementary Fig. 4. Biplots of the first two axis of different multivariate analysis using all assemblages. (a) Principal Coordinate Analysis (PCO) based on Corrected Forbes coefficient, **(b)** PCO based on Bray-Curtis coefficient, **(c)** Non-Metric Multidimensional Scaling Analysis (NMDS) based on Corrected Forbes coefficient, **(d)** NMDS based on Bray-Curtis coefficient. Yellow triangles: faunas from Buenos Aires Province; orange circles: faunas from La Pampa Province (both within Pampean Region); blue squares: faunas from Cuyo and Northwestern regions of Argentina. Abbreviations in Table 1.

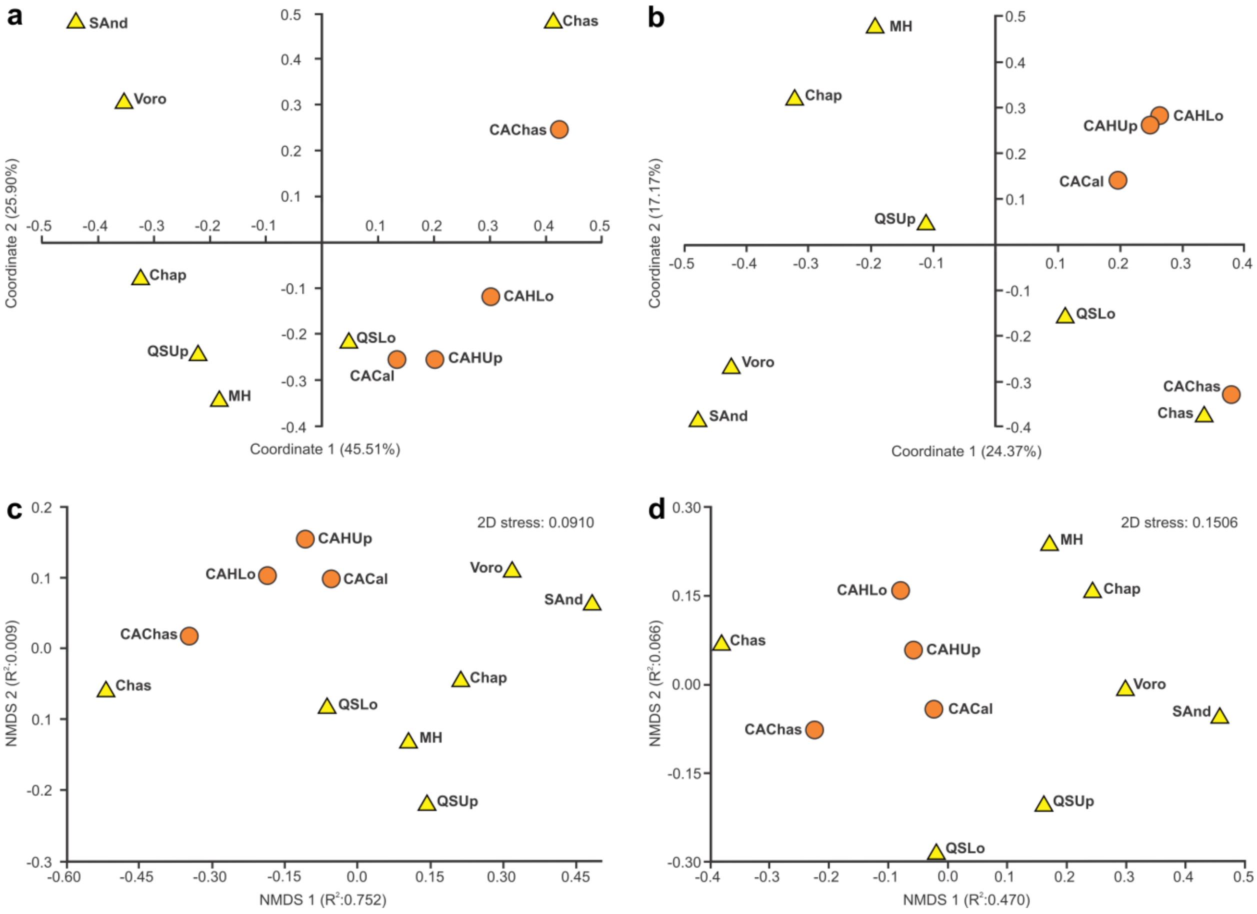

Supplementary Fig. 5. Biplots of the first two axis of different multivariate analysis using assemblages from Pampean Region. (a) Principal Coordinate Analysis (PCO) based on Corrected Forbes coefficient, **(b)** PCO based on Bray-Curtis coefficient, **(c)** Non-Metric Multidimensional Scaling Analysis (NMDS) based on Corrected Forbes coefficient, **(d)** NMDS based on Bray-Curtis coefficient. Yellow triangles: faunas from Buenos Aires Province; orange circles: faunas from La Pampa Province. Abbreviations in Table 1.

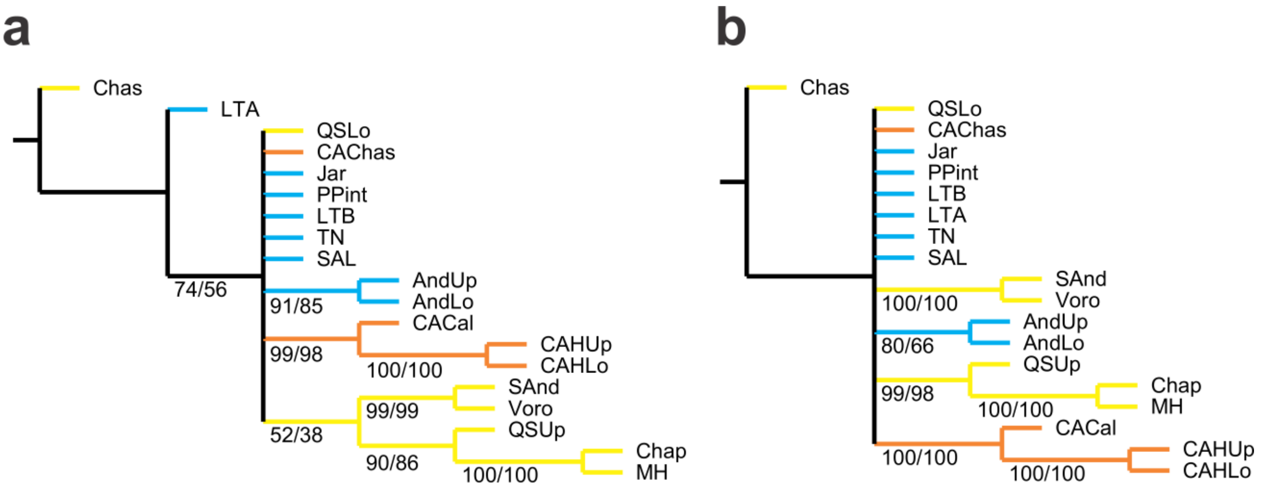

Supplementary Fig. S6. Parsimony Analysis of Endemicity (PAE). **(a)** Strict consensus of four most parsimonious trees of 638 steps, under "standard" parsimony; **(b)** strict consensus of five most parsimonious trees of 677 steps, without reversions (changes from presence to absence were not permitted). Numbers below branches correspond to branch support (frequencies / difference of frequencies, respectively). Yellow lines: faunas from Buenos Aires Province; orange lines: faunas from La Pampa Province (both within Pampean Region); blue lines: faunas from Cuyo and Northwestern regions of Argentina. Abbreviations in Table 1. Trees were obtained with TNT 1.5 (freely available online: http://www.lillo.org.ar/phylogeny/tnt/) and edited with LibreOffice Draw 7.0.3.1 (freely available online: http://www.libreoffice.org/).

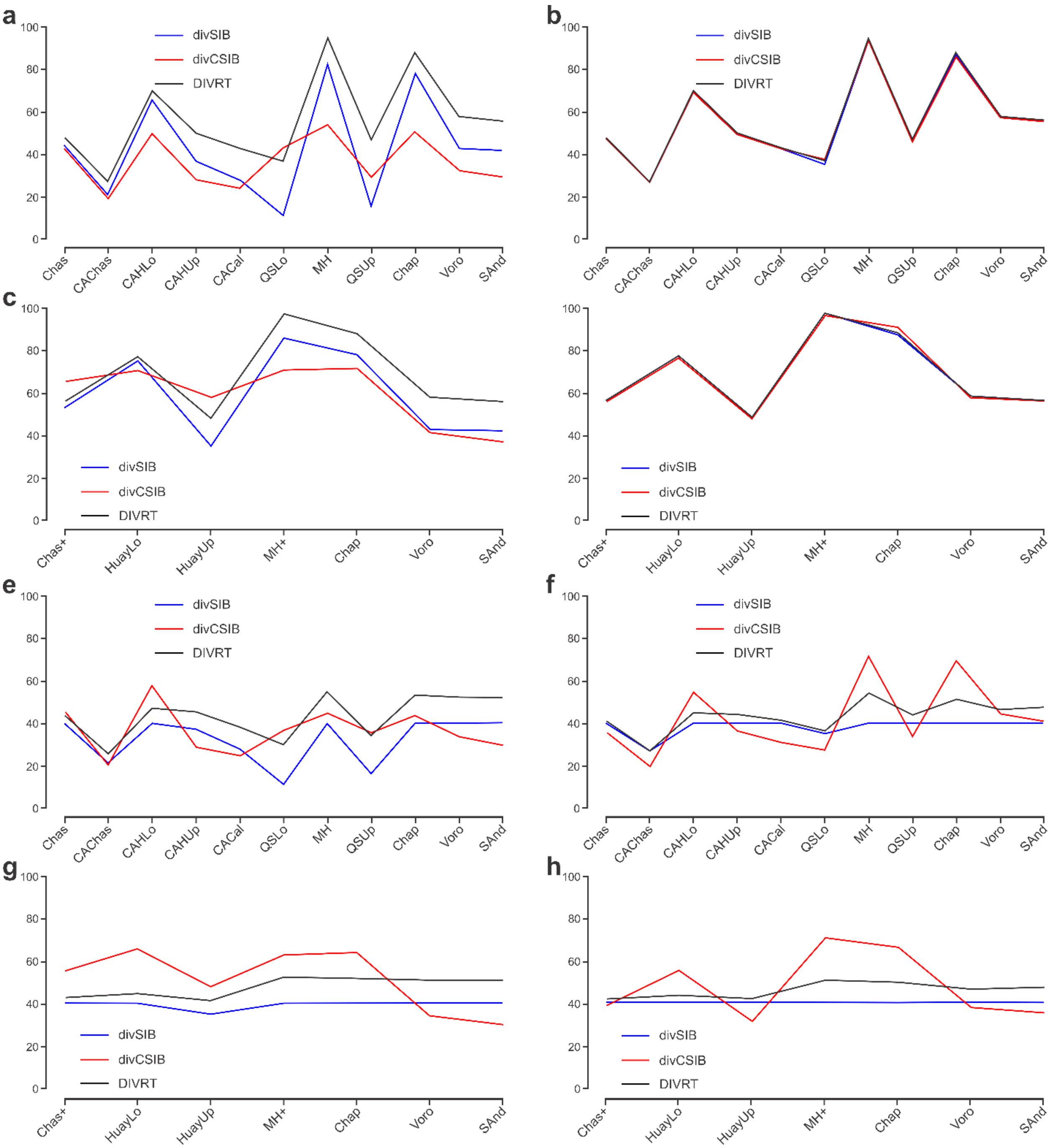

Supplementary Fig. S7. Diversity Rates through different approaches with original (a-d) and resampling (e-h) data. (a, e) Studied faunas approach 1; **(b, f)** studied faunas approach 2; **(c, g)** paired faunas approach 1; **(d, h)** paired faunas approach 2. divCSIB: corrected sample in bin diversity; DIVRT: range-through diversity; divSIB: sample in bin diversity. Chas+: Arroyo Chasicó Fm + Cerro Azul Fm Chasicoan fauna; HuayLo: Cerro Azul Fm lower "Huayquerian" fauna + Cerro Azul Fm upper "Huayquerian" fauna; HuayUp: Cerro Azul Fm Caleufú fauna + lower "Irenean" levels from Quequén Salado River; MH+: Monte Hermoso Fm + upper "Irenean" levels from Quequén Salado River. Abbreviations in Table 1.

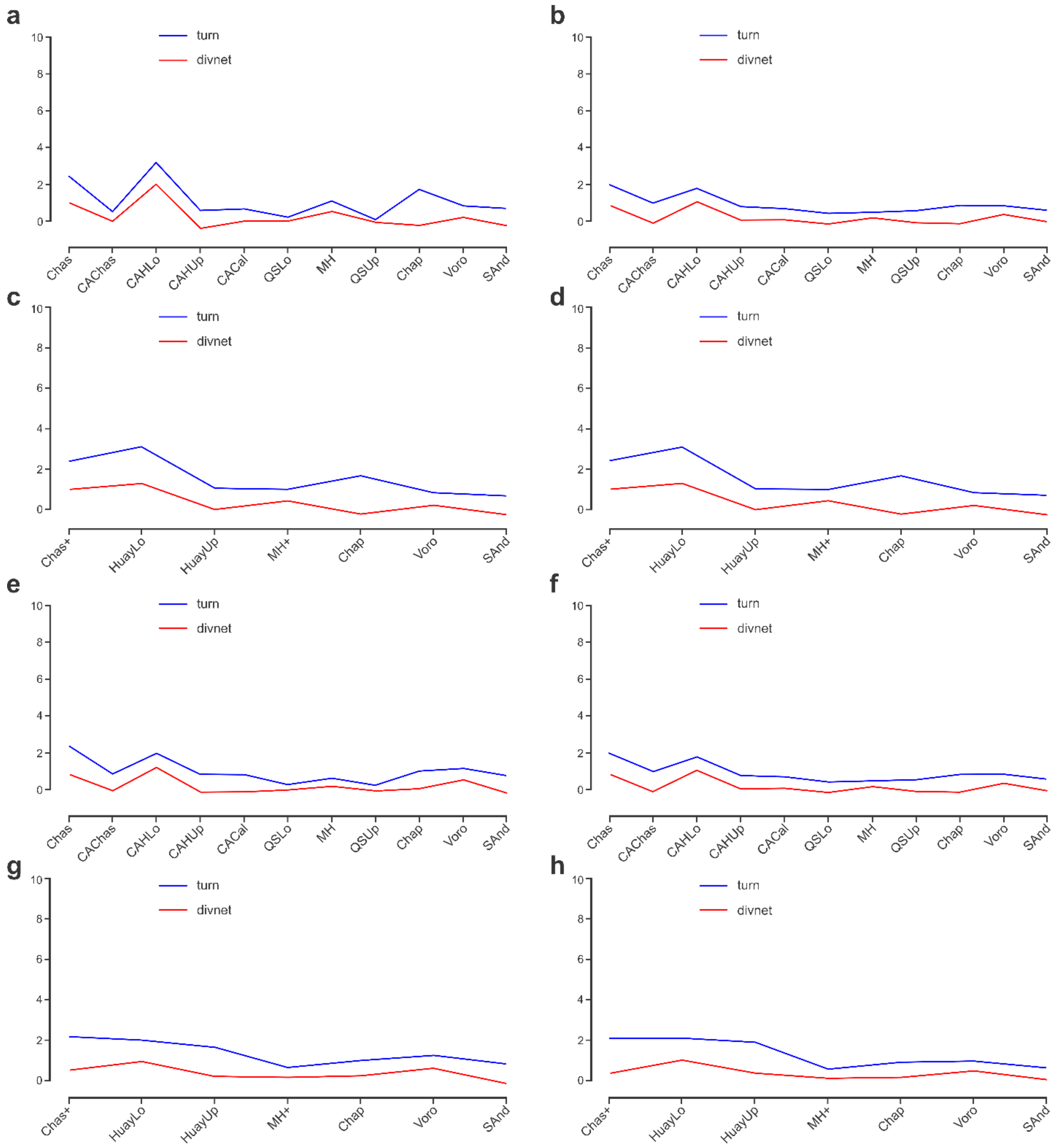

Supplementary Fig. S8. Turnover and Net Diversification Rates through different approaches with original (a-d) and resampling (e-h) data. (a, e) Studied faunas approach 1; **(b, f)** studied faunas approach 2; **(c, g)** paired faunas approach 1; **(d, h)** paired faunas approach 2. divnet: net diversification; turn: turnover rate. Chas+: Arroyo Chasicó Fm + Cerro Azul Fm-Chasicoan fauna; HuayLo: Cerro Azul Fm lower “Huayquerian” fauna + Cerro Azul Fm upper “Huayquerian” fauna; HuayUp: Cerro Azul Fm Caleufú fauna + lower "Irenean" levels from Quequén Salado River; MH+: Monte Hermoso Fm + upper "Irenean" levels from Quequén Salado River. Abbreviations in Table 1.

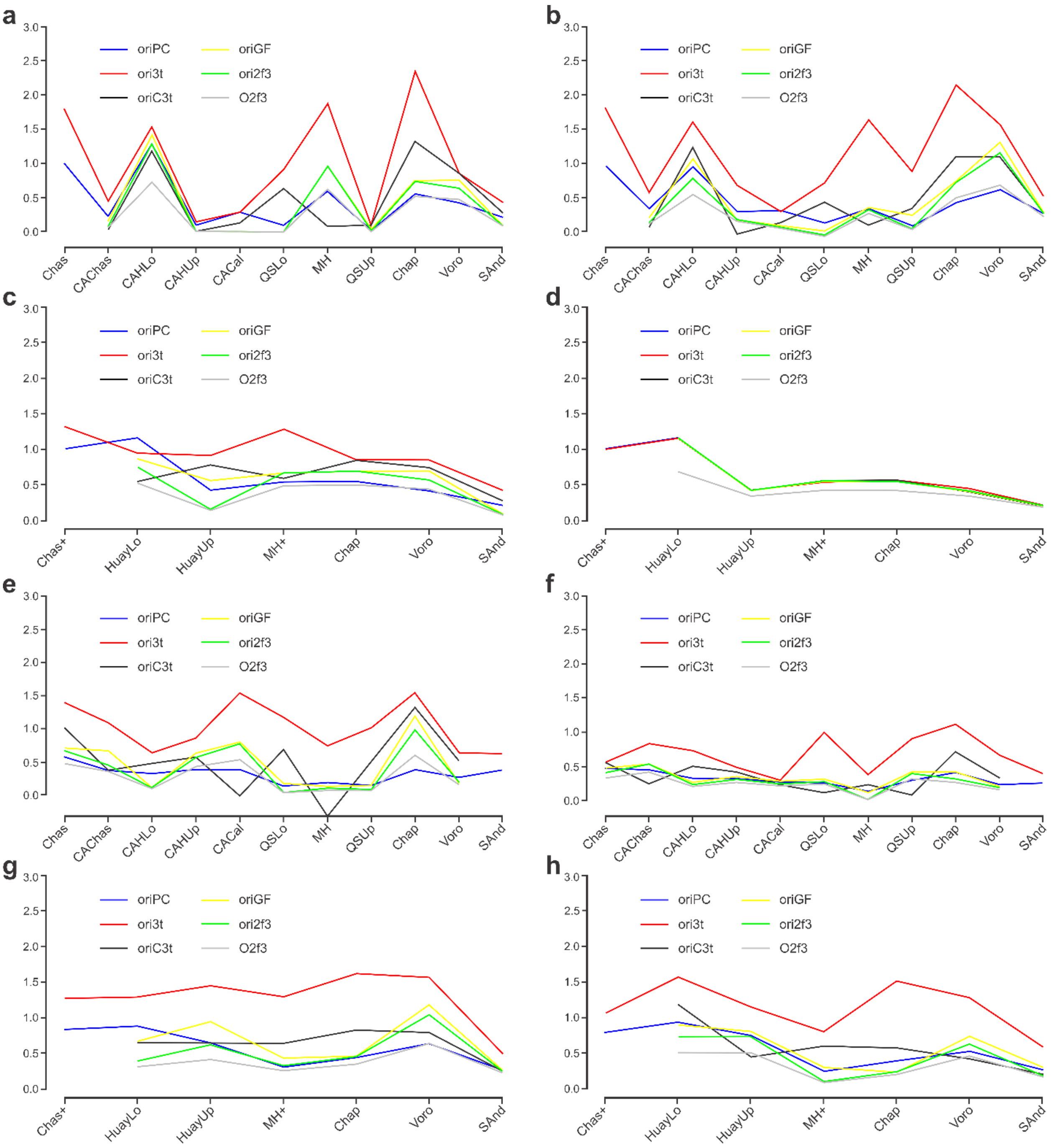

Supplementary Fig. 9. Origination Rates through different approaches with original (a-d) and resampling (e-h) data. (a, e) Studied faunas approach 1; **(b, f)** studied faunas approach 2; **(c, g)** paired faunas approach 1; **(d, h)** paired faunas approach 2. oriPC: per capita origination; ori3t: three-timer origination rates; oriC3t: corrected three-timer origination rates; oriGF: gap filler extinction rates; O2f3: second-for-third origination proportions; ori2f3: second-for-third origination rates. Chas+: Arroyo Chasicó Fm + Cerro Azul Fm Chasicoan fauna; HuayLo: Cerro Azul Fm lower “Huayquerian” fauna + Cerro Azul Fm upper “Huayquerian” fauna; HuayUp: Cerro Azul Fm Caleufú fauna + lower "Irenean" levels from Quequén Salado River; MH+: Monte Hermoso Fm + upper "Irenean" levels from Quequén Salado River. Abbreviations in Table 1.

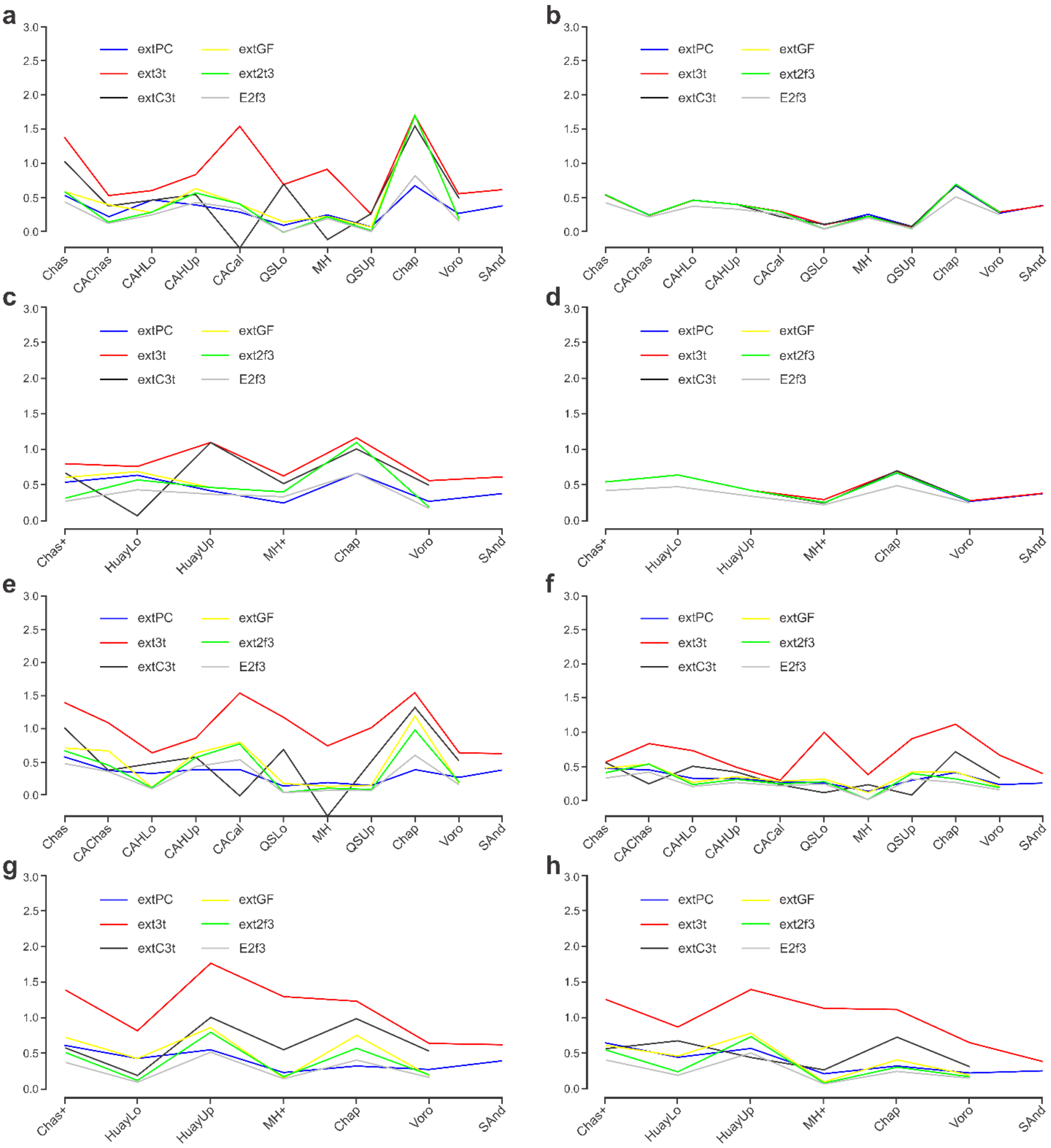

Supplementary Fig. 10. Extinction Rates through different approaches with original (a-d) and resampling (e-h) data. (a, e) Studied faunas approach 1; **(b, f)** studied faunas approach 2; **(c, g)** paired faunas approach 1; **(d, h)** paired faunas approach 2. extPC: per capita extinction; ext3t: three timer extinction rates; extC3t: corrected three-timer extinction rates; extGF: Gap filler extinction rates; E2f3: second for third extinction proportions; ext2f3: second for third extinction rates. Chas+: Arroyo Chasicó Fm + Cerro Azul Fm-Chasicoan fauna; HuayLo: Cerro Azul Fm lower “Huayquerian” fauna + Cerro Azul Fm upper “Huayquerian” fauna; HuayUp: Cerro Azul Fm-Caleufú fauna + lower "Irenean" levels from Quequén Salado River; MH+: Monte Hermoso Fm + upper "Irenean" levels from Quequén Salado River. Abbreviations in Table 1.

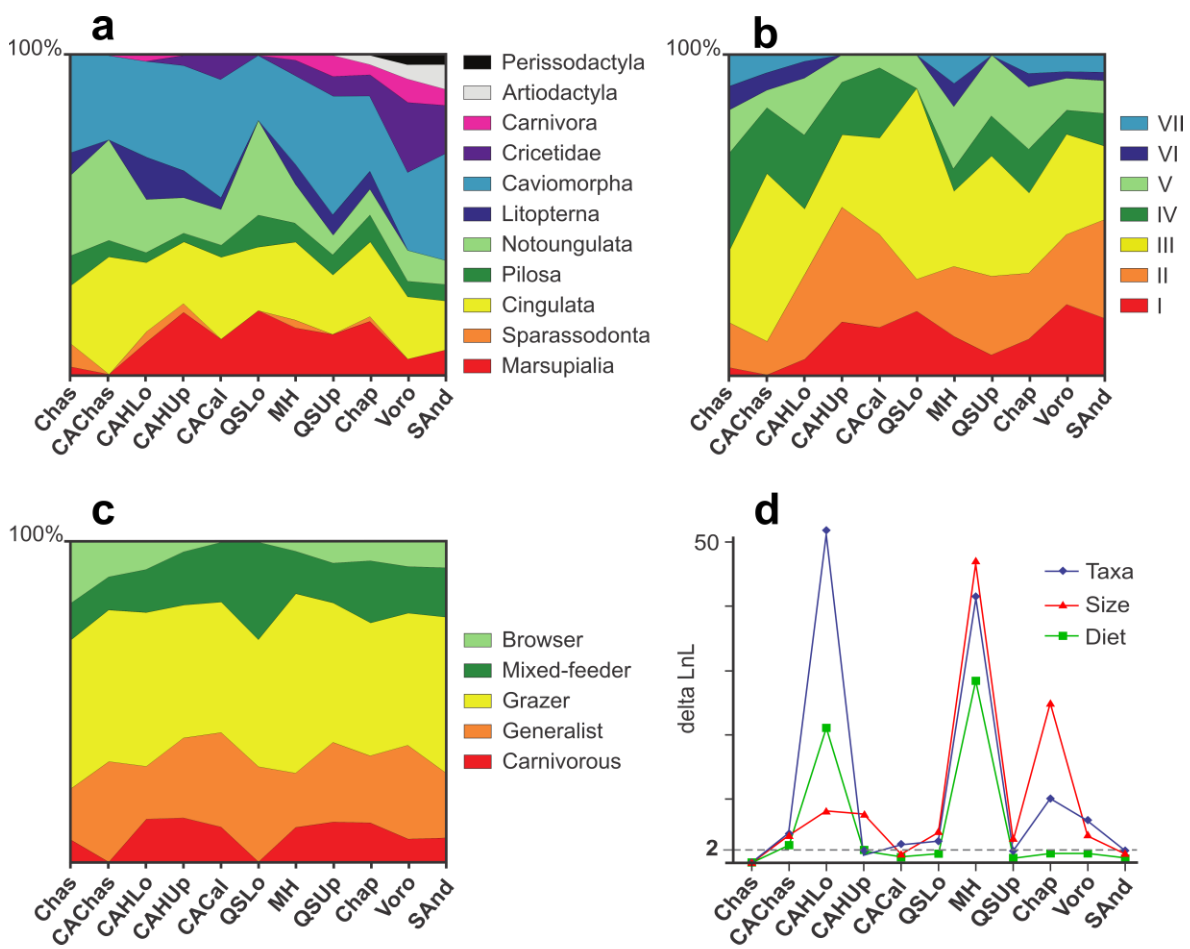

Supplementary Fig 11. Changes in structural parameters of studied faunas through time (approach 1). (a) Proportion of taxonomic groups; **(b)** proportion of body size classes (I, less than 100 g; II, between 100 g and 1 kg; III, 1–10 kg; IV, 10–100 kg; V, 100–500 kg; VI, 500–1000 kg; and VII, more than 1000 kg); **(c)** proportion of diet classes; **(d)** Log-Likelihood difference between successive faunas (significant cutoff [2] is indicated with a dashed line). Abbreviations in Table 1.

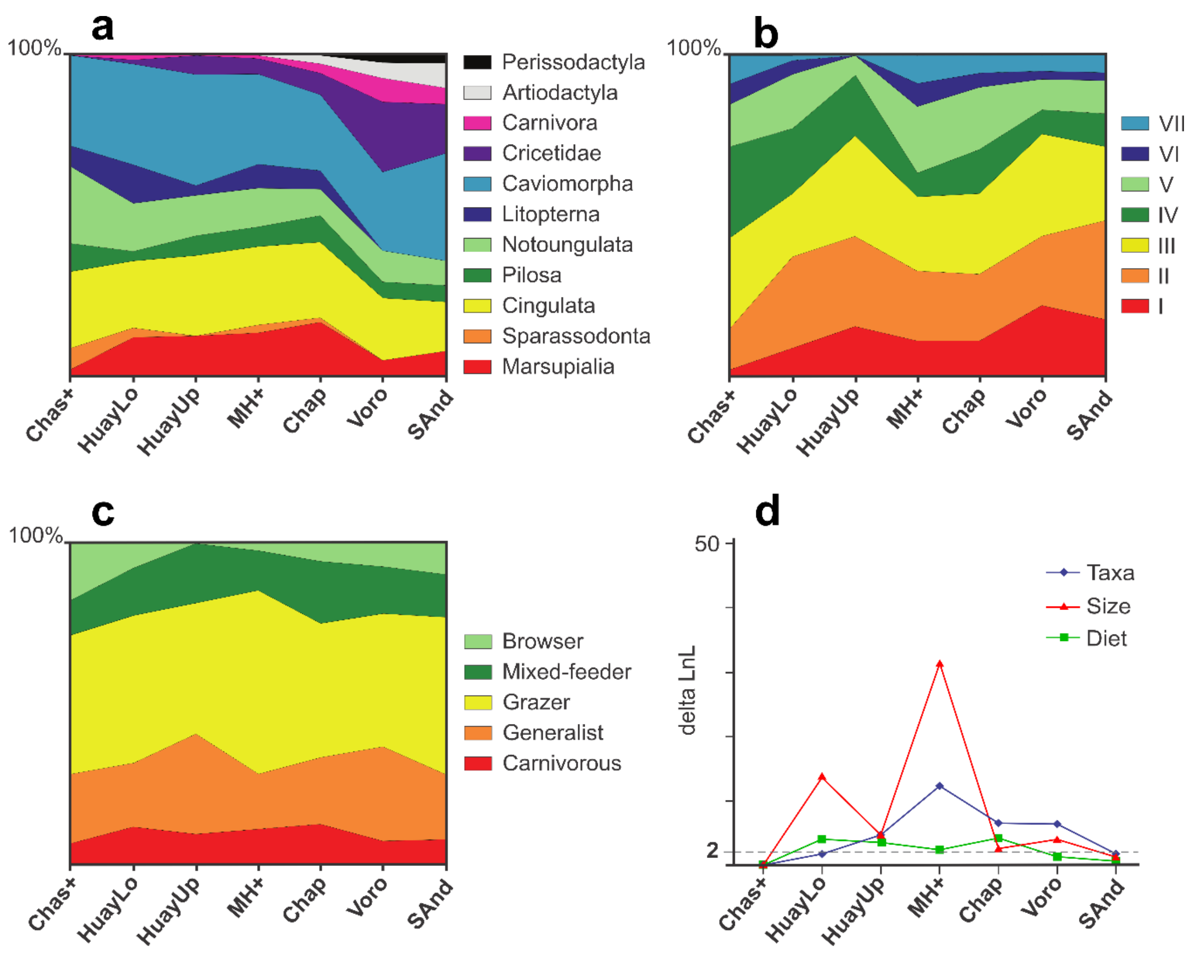

Supplementary Fig 12. Changes in structural parameters of paired faunas through time (approach 1). (a) Proportion of taxonomic groups; **(b)** proportion of body size classes (I, less than 100 g; II, between 100 g and 1 kg; III, 1–10 kg; IV, 10–100 kg; V, 100–500 kg; VI, 500–1000 kg; and VII, more than 1000 kg); **(c)** proportion of diet classes; **(d)** Log Likelihood difference between successive grouped faunas (significant cutoff [2] is indicated with a dashed line). Chas+: Arroyo Chasicó Fm + Cerro Azul Fm-Chasicoan fauna; HuayLo: Cerro Azul Fm lower "Huayquerian" fauna + Cerro Azul Fm upper "Huayquerian" fauna; HuayUp: Cerro Azul Fm Caleufú fauna + lower "Irenean" levels from Quequén Salado River; MH+: Monte Hermoso Fm + upper "Irenean" levels from Quequén Salado River; Chap: Chapadmalal Fm; Voro: Vorohué Fm; SAnd: San Andrés Fm.

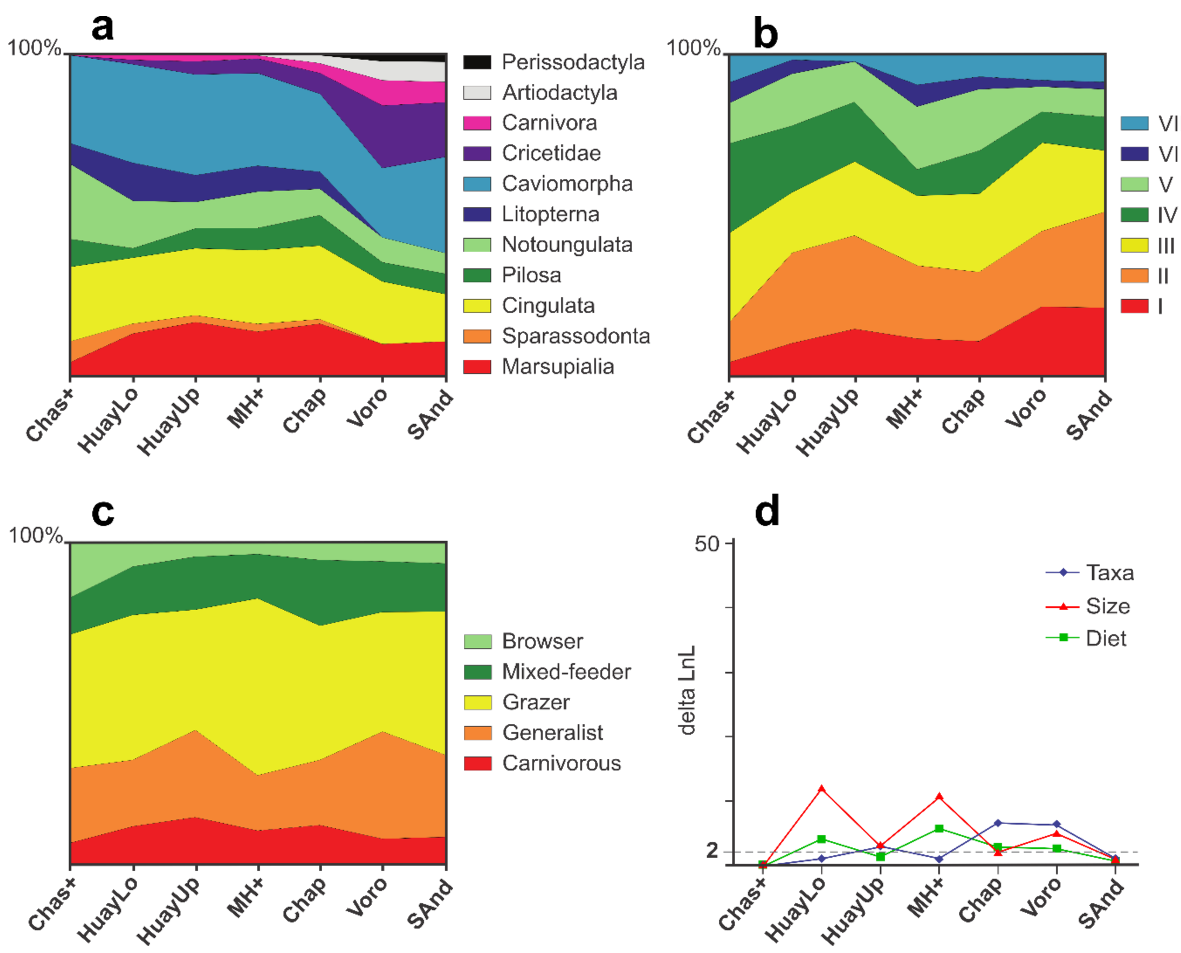

Supplementary Fig 13. Changes in structural parameters of paired faunas through time (approach 2). (a) Proportion of taxonomic groups; **(b)** proportion of body size classes (I, less than 100 g; II, between 100 g and 1 kg; III, 1–10 kg; IV, 10–100 kg; V, 100–500 kg; VI, 500–1000 kg; and VII, more than 1000 kg); **(c)** proportion of diet classes; **(d)** Log Likelihood difference between successive grouped faunas (significant cutoff [2] is indicated with a dashed line). Chas+: Arroyo Chasicó Fm + Cerro Azul Fm-Chasicoan fauna; HuayLo: Cerro Azul Fm lower “Huayquerian” fauna + Cerro Azul Fm upper “Huayquerian” fauna; HuayUp: Cerro Azul Fm Caleufú fauna + lower "Irenean" levels from Quequén Salado River; MH+: Monte Hermoso Fm + upper "Irenean" levels from Quequén Salado River; Chap: Chapadmalal Fm; Voro: Vorohué Fm; SAnd: San Andrés Fm.

Supplementary Table S1. New radioisotopic dates for faunas of the Irene and Chapadmalal Fms (Buenos Aires Province, Argentina). 1# Cascada Grande sample was collected in the upper section of "Irenean", Cascada Grande paleontological locality, Quequén Salado River (-38.626653°, -60.607408°); sampling conducted on the locality's left margin, ca. 20 m downstream from the waterfall, upper part of level 3*sensu* [1]. Two samples (three analyzed aliquots: 2# Quequén Salado, 1# Paso del Halcón, and 2# Paso del Halcón) were collected in Paso del Halcón locality at the lower section of "Irenean", Quequén Salado River (-38.827°, -60.537°); sampling conducted on the locality's left margin, level 2*sensu* [1]. Chapadmalal Fm sample (one aliquot) was taken from the level (or "bank") X of Chapadmalal Fm*sensu* [2] (paleosoil 6 of Playa de Los Lobos Alloformation*sensu* [3, 4]) at Playa La Estafeta (-38.168°, -57.635°). This is the same level as the one previously dated*sensu* [3]. One sample (two analyzed aliquots: 1# APSL P3 and 2# APSL P3) were collected at Playa Las Palomas (-38.149°, -57.615°), from level VI of Chapadmalal Fm*sensu* [2] (paleosoil 3 of the Punta San Carlos Alloformation*sensu* [3, 5]).

**Locality**	**Sample**	**Latitude**	**Longitude**	**Geologic unit and level**	**Plateau Age (SD)**
Cascada Grande	1# Cascada Grande	-38.627º	-60.607º	"Irenean", unit 3	4.33 (0.06) Ma
Paso del Halcón	2# Quequén Salado	-38.827º	-60.537º	"Irenean", unit 2	5.17 (0.08) Ma
Paso del Halcón	1# Paso del Halcón	-38.827º	-60.537º	"Irenean", unit 2	10.5 (0.6) Ma
Paso del Halcón	2# Paso del Halcón	-38.827º	-60.537º	"Irenean", unit 2	18.8 (1.6) Ma
Playa La Estafeta	1# Chapadmalal Fm	-38.168º	-57,635º	Chapadmalal Fm, level X	3.04 (0.06) Ma
Playa Las Palomas	1# APSL P3	-38.149º	-57.615º	Chapadmalal Fm, level VI	3.74 (0.05) Ma
Playa Las Palomas	2# APSL P3	-38.149º	-57.615º	Chapadmalal Fm, level VI	3.98 (0.19) Ma

Supplementary Table S2. **Scores for Maximum Likelihood Appearance Event Ordination (AEO) and multivariate statistics (first two axes) used in the correlation and regression analyses for all relevant assemblages.** CA: Correspondence Analysis; NMDS: Non-Metric Multidimensional Scaling; PCO: Principal Coordinate Analysis; F: analysis based on Corrected Forbes coefficient; Rs: analysis based on Bray-Curtis coefficient; 1: first axis; 2: second axis. Older faunas have a lower position, and vice versa, as established by the AEO score. Abbreviation in Table 1.

Faunas	AEO	CA 1	CA 2	NMDS 1 F	NMDS 2 F	NMDS 1 Rs	NMDS 2 Rs	PCO 1 F	PCO 2 F	PCO 1 Rs	PCO 2 Rs
SAnd	384.5	1.781	-2.285	0.422	0.126	0.419	0.040	0.357	-0.402	0.387	0.383
Voro	367.5	1.495	-1.654	0.294	0.122	0.287	-0.003	0.346	-0.296	0.396	0.289
Chapa	307.5	0.940	0.077	0.207	-0.014	0.258	-0.098	0.354	-0.025	0.396	-0.044
QSUp	282.5	0.535	0.659	0.243	-0.090	0.172	0.145	0.308	-0.005	0.206	0.031
MH	269.5	0.708	0.984	0.144	-0.068	0.219	-0.125	0.279	0.165	0.325	-0.167
CACal	219.5	-0.126	0.615	0.051	0.039	0.034	0.030	0.055	0.066	0.025	-0.142
AndUP	197.5	-0.345	0.881	-0.119	-0.257	-0.067	-0.225	-0.040	0.406	-0.078	-0.351
QSLo	195.5	-0.015	0.447	0.101	0.090	0.050	0.192	0.144	-0.073	0.018	0.133
TN	191.5	-0.319	0.623	0.049	-0.242	-0.060	0.260	0.035	0.354	-0.038	-0.114
AndLo	180.5	-0.399	0.706	-0.099	-0.120	-0.029	-0.113	-0.081	0.265	-0.069	-0.365
CAHUp	161.5	-0.347	0.647	-0.004	-0.002	0.012	-0.018	-0.030	0.170	0.002	-0.267
CAHLo	146.5	-0.551	0.492	-0.060	0.012	0.000	-0.068	-0.153	0.110	-0.024	-0.238
LTB	111.5	-0.906	0.038	-0.220	-0.033	-0.216	0.130	-0.172	-0.021	-0.171	0.121
PPint	106.5	-0.947	-0.074	-0.192	0.005	-0.176	0.060	-0.198	-0.008	-0.235	0.122
Jar	97.5	-0.541	0.412	-0.078	-0.120	-0.044	-0.084	-0.093	0.197	-0.091	-0.234
Sal	78.5	-0.754	0.017	-0.093	0.126	-0.139	-0.052	-0.193	-0.073	-0.212	0.063
CAChas	63.5	-1.216	-0.578	-0.128	0.085	-0.136	0.030	-0.267	-0.120	-0.302	0.176
LTA	48.5	-1.579	-1.115	-0.256	0.163	-0.278	-0.003	-0.330	-0.351	-0.300	0.332
Chas	48.5	-1.910	-1.778	-0.261	0.181	-0.303	-0.098	-0.321	-0.360	-0.235	0.272

Supplementary Table S3. Scores for Maximum Likelihood Appearance Event Ordination (AEO) and multivariate statistics (first two axes) used in the correlation and regression analyses for Pampean Region faunas. AEO: Maximum Likelihood Appearance Event Ordination; CA: Correspondence Analysis; NMDS: Non-Metric Multidimensional Scaling; PCO: Principal Coordinate Analysis; F: analysis based on Corrected Forbes coefficient; Rs: analysis based on Bray-Curtis coefficient; 1: first axis; 2: second axis; Older faunas have a lower position, and vice versa, as established by the AEO score. Abbreviation in Table 1.

Faunas	AEO	CA 1	CA 2	NMDS 1 F	NMDS 2 F	NMDS 1 Rs	NMDS 2 Rs	PCO 1 F	PCO 2 F	PCO 1 Rs	PCO 2 Rs
SAnd	318.5	1.210	-2.145	0.490	0.062	0.455	-0.055	-0.438	0.479	-0.478	-0.391
Voro	299.5	0.983	-1.505	0.318	0.107	0.278	-0.009	-0.352	0.302	-0.425	-0.269
Chapa	239.5	0.691	-0.034	0.214	-0.045	0.216	0.157	-0.324	-0.075	-0.323	0.317
QSUp	212.5	0.447	0.497	0.143	-0.219	0.125	-0.204	-0.219	-0.247	-0.111	0.044
MH	200.5	0.537	0.782	0.106	-0.132	0.135	0.237	-0.183	-0.346	-0.195	0.476
QSLo	150.5	-0.071	0.547	-0.063	-0.084	-0.077	-0.293	0.048	-0.218	0.112	-0.160
CACal	137.5	-0.203	0.772	-0.054	0.098	-0.082	-0.041	0.133	-0.256	0.197	0.141
CAHUp	113.5	-0.387	0.917	-0.107	0.153	-0.120	0.058	0.201	-0.255	0.246	0.264
CAHLo	97.5	-0.621	0.797	-0.184	0.102	-0.143	0.159	0.300	-0.120	0.263	0.282
CAChas	40.5	-1.462	-0.302	-0.345	0.017	-0.304	-0.076	0.422	0.247	0.379	-0.329
Chas	40.5	-2.339	-1.351	-0.517	-0.061	-0.484	0.067	0.412	0.490	0.334	-0.377

Supplementary Table S4. Age of dated faunas regressed against AEO scores and multivariate analyses. AEO: Maximum Likelihood Appearance Event Ordination; CA: Correspondence Analysis; NMDS: Non-Metric Multidimensional Scaling; PCO: Principal Coordinate Analysis; W: whole sample; P: Pampean Region Fauna; F: analysis based on Corrected Forbes coefficient; Rs: analysis based on Bray-Curtis coefficient; a: y-intercept; b: slope; p.: probability; r^2: determinant coefficient; PE%: proportional prediction error of each equation; 1 and 2: first and second axis of multivariate analyses, respectively.

Variable	Axis	a	b	p.	r^2	PE%
AEO P	-	8.620	-0.020	0.01518	0.956	5.202
AEO W	-	9.490	-0.019	0.00000	0.952	3.937
CA P	1	4.718	-1.929	0.00192	0.990	1.503
CA P	2	3.938	0.789	0.70149	0.011	76.450
CA W	1	5.536	-1.879	0.00000	0.899	7.572
CA W	2	5.290	1.357	0.19338	0.049	73.392
NMDS P F	1	5.217	-7.754	0.00203	0.978	5.791
NMDS P F	2	3.270	-4.840	0.46949	0.158	69.841
NMDS P Rs	1	5.190	-8.356	0.00314	0.974	6.494
NMDS P Rs	2	3.796	-2.617	0.78938	0.003	88.233
NMDS W F	1	5.589	-8.054	0.00017	0.778	17.072
NMDS W F	2	6.642	2.096	0.71906	<0.001	173.371
NMDS W Rs	1	5.613	-8.631	0.00012	0.849	10.994
NMDS W Rs	2	6.313	-0.685	0.92644	0.009	141.158
PCO P F	1	5.818	8.286	0.00670	0.966	4.366
PCO P F	2	3.619	-2.883	0.63336	0.023	92.796
PCO P Rs	1	5.863	7.678	0.01971	0.900	10.846
PCO P Rs	2	4.482	-3.459	0.86143	0.061	165.516
PCO W F	1	6.374	-8.444	0.00059	0.870	16.775
PCO W F	2	5.334	2.264	0.49755	0.036	79.260
PCO W Rs	1	5.836	-7.787	0.00048	0.837	12.645
PCO W Rs	2	5.497	-2.447	0.51075	0.040	83.131

Supplementary Table S5. Age estimation for Monte Hermoso Fm (in bold). Min: minimum limit of age estimation; Max: maximum limit of age estimation; Median: median value of all significant estimations (r^2 >0.8) for Age, Min, and Max values; Median PE% <10: median age estimation of equations with PE% lower than 10 (grey cells). AEO: Maximum Likelihood Appearance Event Ordination; CA: Correspondence Analysis; NMDS: Non-Metric Multidimensional Scaling; PCO: Principal Coordinate Analysis; W: whole sample; P: Pampean Region Fauna; F: analysis based on Corrected Forbes coefficient; Rs: analysis based on Bray-Curtis coefficient; r^2: determinant coefficient; PE%: proportional prediction error of each equation; 1: first axis of multivariate analyses.

Variable	Axis	r^2	PE%	Age	Min	Max
CA P	1	0.990	1.503	**3.683**	3.526	4.173
AEO W	-	0.952	3.937	**4.373**	2.763	4.741
PCO P F	1	0.966	4.366	**4.300**	3.753	4.611
AEO P	-	0.956	5.202	**4.572**	4.167	5.227
NMDS P F	1	0.978	5.791	**4.397**	4.082	4.875
NMDS P Rs	1	0.974	6.494	**4.063**	3.728	4.590
CA W	1	0.899	7.572	**4.206**	3.432	4.837
PCO P Rs	1	0.900	10.846	**4.368**	3.622	4.716
NMDS W Rs	1	0.849	10.994	**3.719**	3.110	4.657
PCO W Rs	1	0.837	12.645	**3.304**	2.518	4.045
PCO W F	1	0.870	16.775	**4.014**	2.670	4.585
NMDS W F	1	0.778	17.072	**4.429**	3.439	5.438
Median				**4.253**	3.483	4.687
Median PE% <10				**4.300**	3.728	4.741

Supplementary Table S6. Age estimation for Cerro Azul assemblages (in bold). CACal: Cerro Azul Fm, Caleufú fauna; CAHUp: Cerro Azul Fm, lower "Huayquerian" fauna; CAHLo: Cerro Azul Fm, upper "Huayquerian" fauna; CAChas: Cerro Azul Fm, Chasicoan fauna. Min: minimum limit of age estimation; Max: maximum limit of age estimation; Median: median value of all significant estimations ($r^2 > 0.8$) for Age, Min, and Max values; Median PE% <10: median age estimation of equations with PE% lower than 10 (grey cells). AEO: Maximum Likelihood Appearance Event Ordination; CA: Correspondence Analysis; NMDS: Non-Metric Multidimensional Scaling; PCO: Principal Coordinate Analysis; W: whole sample; P: Pampean Region Fauna; F: analysis based on Corrected Forbes coefficient; Rs: analysis based on Bray-Curtis coefficient; r^2: determinant coefficient; PE%: proportional prediction error of each equation; 1: first axis of multivariate analyses.

				**CACal**			**CAHUp**			**CAHLo**			**CAChas**		
**Variable**	**Axis**	**r^2**	**PE%**	**Age**	**Min**	**Max**	**Age**	**Min**	**Max**	**Age**	**Min**	**Max**	**Age**	**Min**	**Max**
CA P	1	0.990	1.503	**5.109**	4.992	5.473	**5.464**	5.358	5.797	**5.915**	5.821	6.208	**7.538**	7.490	7.687
AEO W	-	0.952	3.937	**5.323**	4.073	5.756	**6.424**	5.594	6.935	**6.709**	5.987	7.239	**8.285**	8.162	8.925
PCO P F	1	0.966	4.366	**6.921**	5.694	7.066	**7.486**	6.113	7.596	**8.305**	6.719	8.363	**9.312**	7.465	9.307
AEO P	-	0.956	5.202	**5.844**	5.431	6.803	**6.329**	5.912	7.404	**6.652**	6.233	7.804	**7.802**	7.376	9.230
NMDS P F	1	0.978	5.791	**5.637**	5.115	5.992	**6.045**	5.456	6.360	**6.648**	5.959	6.903	**7.895**	6.999	8.027
NMDS P Rs	1	0.974	6.494	**5.871**	5.202	6.214	**6.192**	5.464	6.502	**6.382**	5.619	6.673	**7.733**	6.720	7.886
CA W	1	0.899	7.572	**5.773**	4.397	6.896	**6.187**	4.652	7.440	**6.572**	4.889	7.945	**7.821**	5.659	9.586
PCO P Rs	1	0.900	10.846	**7.374**	5.599	8.060	**7.755**	5.849	8.484	**7.884**	5.934	8.628	**8.774**	6.520	9.619
NMDS W Rs	1	0.849	10.994	**5.321**	4.106	6.953	**5.507**	4.222	7.220	**5.615**	4.290	7.375	**6.790**	5.020	9.059
PCO W Rs	1	0.837	12.644	**5.642**	4.107	6.850	**5.819**	4.227	7.052	**6.022**	4.365	7.294	**8.184**	5.835	9.879
PCO W F	1	0.870	16.775	**5.913**	4.064	6.599	**6.625**	4.586	7.354	**7.664**	5.349	8.455	**8.630**	6.058	9.479
NMDS W F	1	0.778	17.072	**5.182**	3.869	6.842	**5.621**	4.119	7.088	**6.075**	4.379	7.717	**6.618**	4.689	8.470
Median				**5.708**	4.695	6.823	**6.190**	5.407	7.154	**6.610**	5.720	7.546	**7.858**	6.620	9.145
Median PE% <10				**5.773**	5.115	6.214	**6.192**	5.464	6.935	**6.648**	5.959	7.239	**7.821**	7.376	8.925

Supplementary Table S7. Correlations (Spearmann ρ) between latitude-longitude and AEO and first two axes of multivariate analysis scores for Whole Sample and Pampean Region faunas. AEO: Maximum Likelihood Appearance Event Ordination; CA: Correspondence Analysis; NMDS: Non-Metric Multidimensional Scaling; PCO: Principal Coordinate Analysis; F: analysis based on Corrected Forbes coefficient; Rs: analysis based on Bray-Curtis coefficient; 1 and 2: first and second axis of multivariate analyses, respectively. Significant correlation values under False Discovery Rate are in red.

**Whole Sample**	AEO	CA1	CA2	PCO 1 F	PCO 2 F	PCO 1 Rs	PCO 2 Rs	NMDS 1 F	NMDS 2 F	NMDS 1 Rs	NMDS 2 Rs
Latitude	0.4122	0.4521	-0.0932	0.5330	0.3720	0.5400	0.1117	0.4776	-0.4529	0.5488	0.3747
p.	0.0795	0.3465	0.4406	0.0387	0.0515	0.0150	0.1140	0.0188	0.1168	0.0170	0.6489
Longitude	-0.6542	-0.6649	0.1073	-0.7194	-0.2559	-0.7810	0.0677	-0.6939	0.3298	-0.7458	-0.2076
p.	0.0024	0.3465	0.4406	0.0010	0.1679	0.0002	0.3938	0.0005	0.2902	0.0001	0.7830

**Pampean Region**	AEO	CA1	CA2	PCO 1 F	PCO 2 F	PCO 1 Rs	PCO 2 Rs	NMDS 1 F	NMDS 2 F	NMDS 1 Rs	NMDS 2 Rs
Latitude	0.3151	0.3144	-0.2597	-0.3326	0.0501	-0.3508	-0.0957	0.2141	-0.7882	0.3144	-0.1093
p.	0.3453	0.3465	0.4406	0.3176	0.8837	0.2902	0.7796	0.5272	0.0040	0.3465	0.7490
Longitude	-0.8356	-0.8064	0.4784	0.8519	-0.3235	0.8428	0.0319	-0.7608	0.2323	-0.8064	0.0000
p.	0.0014	0.0027	0.1367	0.0009	0.3319	0.0011	0.9258	0.0065	0.4918	0.0027	1.0000

Supplementary Dataset S1 (separate file). Taxa and localities data matrix.

Supplementary Dataset S2 (separate file). Databases of taxonomic groups, diet, and body size of each taxon per every fauna and paired faunas of the Pampean Region. .

References

1. Pardiñas, U. F. J. *et al*. A controversial unit within the argentine Neogene: The "Irenean" fauna. *Ameghiniana* **54,** 655–680 (2017).
2. Kraglievich, J. L. El perfil geológico de Chapadmalal y Miramar. Provincia de Buenos Aires. *Rev. Mus. Municip. Cs. Nat. Tradicional Mar del Plata* **1,** 8–37 (1952).
3. Schultz, P. *et al.*, A 3.3 Ma impact in Argentina and possible consequences. *Science* **282,** 2061–2063 (1998).
4. Zárate, M. A. *et al.* Geology and geochronology of type Chasicoan (Late Miocene) mammal-bearing deposits of Buenos Aires (Argentina). *J. S. Am. Earth Sci.* **23,** 81–90 (2007).
5. Zárate, M. A., & Fasano, J. The Plio-Pleistocene record of the central eastern Pampas, Buenos Aires province, Argentina: The Chapadmalal case study. *Palaeogeogr. Palaeoclimatol. Palaeoecol.* **72,** 27–52 (1989).
